# Insights Into the Structure-Function Relationships of Dimeric C3d Fragments

**DOI:** 10.3389/fimmu.2021.714055

**Published:** 2021-08-09

**Authors:** Ayla A. Wahid, Rhys W. Dunphy, Alex Macpherson, Beth G. Gibson, Liudmila Kulik, Kevin Whale, Catherine Back, Thomas M. Hallam, Bayan Alkhawaja, Rebecca L. Martin, Ingrid Meschede, Maisem Laabei, Alastair D. G. Lawson, V. Michael Holers, Andrew G. Watts, Susan J. Crennell, Claire L. Harris, Kevin J. Marchbank, Jean M. H. van den Elsen

**Affiliations:** ^1^Department of Biology and Biochemistry, University of Bath, Bath, United Kingdom; ^2^UCB Pharma, Slough, United Kingdom; ^3^Translational and Clinical Research Institute, Newcastle University, Newcastle-upon-Tyne, United Kingdom; ^4^Division of Rheumatology, University of Colorado, Aurora, CO, United States; ^5^Department of Pharmacy and Pharmacology, University of Bath, Bath, United Kingdom; ^6^Centre for Therapeutic Innovation, University of Bath, Bath, United Kingdom

**Keywords:** complement, B cell, tolerance, C3d dimers, X-ray crystal and molecular structure

## Abstract

Cleavage of C3 to C3a and C3b plays a central role in the generation of complement-mediated defences. Although the thioester-mediated surface deposition of C3b has been well-studied, fluid phase dimers of C3 fragments remain largely unexplored. Here we show C3 cleavage results in the spontaneous formation of C3b dimers and present the first X-ray crystal structure of a disulphide-linked human C3d dimer. Binding studies reveal these dimers are capable of crosslinking complement receptor 2 and preliminary cell-based analyses suggest they could modulate B cell activation to influence tolerogenic pathways. Altogether, insights into the physiologically-relevant functions of C3d(g) dimers gained from our findings will pave the way to enhancing our understanding surrounding the importance of complement in the fluid phase and could inform the design of novel therapies for immune system disorders in the future.

## Introduction

Activation of the central complement component C3 (~1 mg mL^-1^) to C3a and C3b by classical/lectin (C4bC2a) or alternative (C3bBb) pathway C3 convertases plays an essential role in the generation of complement-mediated defence mechanisms against invading microbial pathogens. While the circulating C3a anaphylatoxin is involved in inducing inflammatory immune responses, C3b (reported (1) normal plasma concentration: ~210 ng mL^-1^ but levels are markedly higher on infection and under certain disease conditions although its short half-life (< 2 min) makes accurate measurements difficult) facilitates opsonophagocytosis and clearance of immune complexes through thioester-mediated opsonisation of primary amine- or hydroxyl-containing antigenic and self surfaces. Attachment of multiple copies of C3b and its breakdown products to antigenic surfaces in this way can result in C3d-complement receptor 2 (CR2/CD21) and antigen-B cell receptor (BCR) co-ligation which generates co-stimulatory signals for B cell activation in a C3d copy-dependent manner (2, 3) and has been widely explored in vaccine design (4–9).

Structure determination of native C3, C3b and C3c has provided crucial insights into the mechanistic basis behind the activation of C3 to C3b (10, 11) while complexes of C3b with factor I (FI) and the short consensus repeat (SCR) domains 1-4 of its cofactor factor H (FH_1-4_) have revealed the processes through which C3b is proteolytically cleaved into its successive opsonin fragments iC3b and C3dg (12) [normal plasma concentration: < 5.3 µg mL^-1^, half-life: 4 hours (13)]. Crystal structures have also shed light upon the molecular basis underlying the thioester-mediated attachment of C3d to antigenic surfaces (14), provided explanations of how the interactions of C3d with its receptors [CR2 (15) and CR3 (16)] facilitate the recognition of opsonised antigens, and the mechanisms by which pathogens such as *Staphylococcus aureus* utilise C3d-binding proteins [e.g. Sbi (17), Efb-C (18, 19) and Ecb/Ehp (20, 21)] to inhibit these interactions and evade the immune system. Furthermore, complexes of C3d with FH SCR domains 19 and 20, which also bind host surface polyanionic markers such as glycosaminoglycans and sialic acids, have been pivotal in understanding the regulatory measures in place to protect host tissues against the indiscriminate attachment of C3d to self versus non-self surfaces (22, 23).

However, while these seminal structural studies alongside an abundance of functional investigations have advanced our knowledge surrounding the interactions of C3 fragments with self and non-self surfaces at a molecular level, our understanding of the structural and functional aspects of fluid phase C3 activation products remains incomplete. During activation in the fluid phase, the majority of C3 molecules do not covalently attach to surface-exposed hydroxyl- or amine-nucleophiles but instead the highly reactive Cys–Gln thioester moiety within the thioester-containing C3d domain (TED) of C3 undergoes hydrolysis resulting in the generation of C3(H_2_O) and formation of the C3(H_2_O)Bb alternative pathway (AP) C3 convertase. Of the C3b generated by these fluid phase or surface-associated convertases, only approximately 10% is deposited onto reactive surfaces (24), leaving the remaining 90% to react with water wherein exposure of the cysteine free sulfhydryl can lead to dimerisation of C3b and its subsequent breakdown products iC3b and C3d(g). Evidence of these dimers has been demonstrated *via* visualisation of C3b generated from trypsin digestion of serum-derived C3 under non-reducing conditions (25) and in C3dg preparations purified from human serum following ‘aging’ at 37°C for 7 days (26).

C3b dimers, formed either by disulphide bonds or *via* other, undefined interactions, have also been found to bind CR1 with 25-fold higher affinity than monomeric C3b (25), induce histaminase release from human polymorphonuclear leukocytes (26), serve as binding platforms for factor B fragment Bb during formation of AP C5 convertases (27, 28) and act as potent AP activators in complex with IgG (29). In addition, dimers of C3dg have been isolated from C3-activated human serum following omission of N-ethylmaleimide (30) and the propensity of recombinant C3d to dimerise has been reported previously (31, 32). A crystal structure of dimeric C3dg purified from rat serum (33) provides further crucial evidence of the endogenous existence of these dimers. However, aside from this severely truncated C3dg dimer which is believed to have undergone proteolytic truncation during the crystallisation process (34), there is currently a gap in knowledge surrounding the structural significance of disulphide-linked dimers of C3 fragments as the thioester cysteine sulfhydryl is routinely removed prior to structural analyses. For instance, the free cysteine of C3b has been reacted with iodoacetamide prior to structural determination (11, 35, 36) and the vast majority of C3d constructs used for structural studies to date have harboured  a cysteine to alanine substitution in order to prevent dimerisation (14, 15, 22, 23).

In this study we therefore aimed to delineate the molecular details and explore the functional significance of dimeric human C3 fragments that can form following activation of C3 in the fluid phase. We provide confirmatory evidence showing the formation of disulphide-linked C3b dimers derived from serum-derived C3 and subsequently present the first crystal structure of a human C3d dimer at 2.0 Å resolution where dimerisation is mediated by disulphide linkage of the thioester cysteine residues. Through surface plasmon resonance (SPR) binding studies and preliminary cell experiments using mouse splenocytes and human peripheral blood mononuclear cells (PBMC) we show how dimeric C3d crosslinks surface-bound CR2 and could modulate B cell activation to potentially influence tolerance mechanisms. In the future, a deeper understanding of these newly-discovered physiologically-relevant roles of C3 fragment dimers could inform the design of autoimmune therapies and help to further elucidate the significance of complement in the fluid phase as it interacts with cells of the adaptive immune system and beyond.

## Materials And Methods

### Purification and Mild Trypsin Proteolysis of Human C3

C3 was purified from human serum by PEG precipitation (37), by slowly mixing the serum with PEG 4000 (in precipitation buffer: 100 mM sodium phosphate, 150 mM NaCl, 15 mM EDTA, 0.5 mM PMSF, pH 7.4) to a final concentration of 5% and then incubating the mixture on ice for 30 mins. After centrifugation, the supernatant was retained, and the process was repeated using PEG 4000 at a final concentration of 12%. The resulting pellet was resuspended in binding buffer and purified by weak anion exchange chromatography (column: 1 mL HiTrap DEAE Sepharose FF (Cytiva), binding buffer: 25 mM potassium phosphate, 5 mM EDTA, 50 mM EACA, pH 7.0, elution buffer: 25 mM potassium phosphate, 5 mM EDTA, 50 mM EACA, 300 mM NaCl, pH 7.0).

The C3 containing fractions were subsequently pooled and 100 µg was digested with Trypsin Gold protease (Promega) at 37°C for 2 mins before being quenched with 3% (w/w) soybean trypsin inhibitor (Sigma Aldrich). An additional t=0 sample was prepared by adding trypsin and trypsin inhibitor at the same time to a sample containing 10 µg C3 before the incubation. The digested protein was then incubated at 18°C for 2 hours, with timepoints taken every 15 mins and analysed using reducing and non-reducing tris-acetate SDS-PAGE.

For western blot analyses, PVDF membrane was initially washed in methanol and then soaked in western blot transfer buffer (methanol-free, Pierce) along with the gel and filter pads. The proteins from the SDS-PAGE gel were then blotted onto the PVDF membrane using a G2 semi-dry fast blotter (Pierce). After the membrane was blocked and subsequently washed, the immunodetection steps were completed on a SNAP id 2.0 western blotting system (Merck Millipore) according to manufacturer’s instructions. The antibodies used include a polyclonal rabbit anti-C3d (Dako) and a polyclonal goat anti-rabbit IgG (H+L) HRP conjugated (Invitrogen). To detect the HRP conjugated antibody, the membrane was incubated with ECL prime western blotting substrate (Amersham) and then imaged on a Fusion SL (VILBER) by chemiluminescence with molecular weight markers highlighted using a WesternSure pen (LI-COR).

### Expression and Purification of Recombinant Proteins

The DNA sequence of human C3d (residues 1-310) comprised of C3 residues 996-1303 (pre-pro C3 numbering) with a Cys to Ala mutation at position 17(C3d)/1010 (pre-pro C3) (C3d^17A^) was previously cloned into the pET15b expression plasmid (14). To reproduce the wild-type sequence, the Ala at position 17 of the C3d^17A^ construct was reverted back to a Cys (C3d^17C^) using site-directed mutagenesis. Both C3d constructs were expressed in the *Escherichia coli* BL21(DE3) (Sigma Aldrich) or Shuffle T7 (NEB) cell lines and purified using cation exchange (column: HiTrap SP HP [GE Healthcare], binding buffer: 50 mM MES pH 5.5, elution buffer: 50 mM MES, 500 mM NaCl pH 5.5) followed by size exclusion chromatography (column: HiLoad 16/600 Superdex 200 prep grade [GE Healthcare], buffer: 20 mM Tris, 150 mM NaCl pH 7.4).

The human CR2(SCR1-4)-Fc and FH_19-20_ constructs used in surface plasmon resonance experiments were expressed in Chinese hamster ovary (CHO) cells or *Pichia pastoris* respectively and purified as described previously [CR2(SCR1-4)-Fc (8); FH_19-20_ (38)]. The monomeric state of FH_19-20_ was confirmed using analytical ultracentrifugation (**Supplementary Figure S9**).

### Crystallisation, Data Collection and Structure Determination of Dimeric C3d^17C^

Crystallisation was performed at 18°C using the hanging drop vapour diffusion method. A 15 mg mL^-1^ (432 µM) C3d^17C^ solution was subjected to a grid screen containing 100 mM Tris pH 8, 50-300 mM NaCl and 16-26% PEG 4000. Crystals appeared in the condition containing 100 mM Tris pH 8, 200 mM NaCl, 24% PEG 4000, were mounted on loops, flash-frozen in liquid nitrogen and X-ray diffraction data collected on the IO4 beamline at the Diamond Light Source synchrotron (Oxfordshire, UK) (see **Supplementary Table S1** for data collection statistics). Integration of Dectris PILATUS 6M pixel detector diffraction images and data reduction were performed using Xia2-DIALS and AIMLESS, respectively. The automated BALBES pipeline and COOT were used for molecular replacement and model building. Refinement was carried out in REFMAC and Phenix (refinement statistics can be found in **Supplementary Table S2**) and UCSF Chimera was used for superpositioning and generation of images. The structure of the C3d^17C^ dimer is available in the protein data bank (PDB) with the following accession code: 6RMT.

### Synthesis and Characterisation of N,N’-(propane-1,3-diyl) bis(2-bromoacetamide) Linker

      
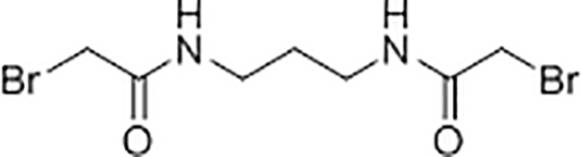


A solution of K_2_CO_3_ (5.92 g, 42.8 mmol) in water (21 mL) was added to a solution of 1,3-diaminopropane (1.06 g, 14.3 mmol) in chloroform (35 mL) at 5°C with stirring. A solution of bromoacetyl bromide (8.65 g, 42.8 mmol) in anhydrous chloroform (15 mL) was then added dropwise to the mixture and the reaction was left stirring at room temperature for 18 hours. The resultant precipitate was filtered, washed with water (6 x 10 mL), and dried under vacuum to yield *N,N’*-(propane-1,3-diyl)bis(2-bromoacetamide) as a white solid (2.45 g, 55%). Subsequent characterisation of the linker was performed using ^1^H-NMR (**Supplementary Figure S4A**) and ^13^C-NMR (**Supplementary Figure S4B**). High resolution electrospray ionisation time-of-flight mass spectrometry m/z: [M + Na]^+^ calculated for C_7_H_12_Br_2_N_2_O_2_Na = 338.9143 Da, 338.9143 Da was observed.

### Production, Purification and Characterisation of Chemically-Linked C3d Dimers

For the generation of chemically-linked C3d^17C^ dimers, small-scale trials were performed involving combination of C3d^17C^ with the *N,N’*-(propane-1,3-diyl)bis(2-bromoacetamide) linker in 0.1 M Tris, 0.15 M NaCl, 5 mM EDTA pH 7.5 at 0.55, 0.75 or 1.0 molar equivalences. Following overnight incubation at room temperature (21°C), linker-mediated C3d^17C^ dimerisation was confirmed using reducing SDS-PAGE and electrospray time-of-flight mass spectrometry (**Supplementary Figure S5**). A larger scale reaction at 0.75 molar equivalence (3.75 mg C3d^17C^, 0.026 mg linker) was subsequently carried out as described above and subjected to size exclusion chromatography to separate the chemically-linked dimeric C3d^17C^ from monomeric C3d^17C^ (**Supplementary Figures S6A, B**). Particle size analysis yielded a single species (**Supplementary Figure S6C**), analytical ultracentrifugation confirmed the dimeric state of the chemically-linked C3d^17C^ (**Supplementary Figure S6D**) and both biophysical techniques showed a lack of aggregate formation. Chemically-linked dimeric C3d^17C^ was subsequently digested with trypsin (Sigma Aldrich) (1:50 ratio) at 37°C over a time course (**Supplementary Figure S6E**). The digestion reaction was stopped by addition of a trypsin inhibitor (Sigma Aldrich) (1:2 ratio). Electrospray ionization time-of-flight mass spectrometry of the trypsin-digested dimeric C3d^17C^ fragments followed by analysis using the Masshunter Qualitative Analysis and BioConfirm (Agilent) software packages was used to confirm chemical linkage at position 17C of C3d (**Supplementary Table S3**) and the presence of an intact internal disulphide bond (**Supplementary Table S4**).

### Surface Plasmon Resonance

All surface plasmon resonance experiments were performed on a Biacore S200 sensor (GE Healthcare) at 25°C with HBST (10 mM HEPES, 150 mM NaCl, 0.005% Tween-20, pH 7.4) used as the running buffer. CR2-Fc and FH_19-20_ were prepared in 10 mM sodium acetate pH 5 and immobilised at 300 RU (CR2-Fc: 20 µM, FH_19-20_: 240 µM) to different flow cells of CM5 chips (GE Healthcare) using standard amine coupling involving preparation of the dextran matrix with 100 mM N-hydroxysuccinimide (NHS) and 40 mM 1-ethyl-3-(dimethylaminopropyl) carbodiimide (EDC) followed by quenching with 1 M ethanolamine-HCl pH 8.5. Monomeric C3d^17A^ and chemically-linked dimeric C3d^17C^ used as analytes were prepared to a fixed concentration, serially diluted in HBST and injected in duplicate. 10 mM sodium acetate, 1 M NaCl pH 4 was used as the regeneration buffer but could not regenerate the chip surface of the highly avid interactions between dimeric C3d^17C^ and CR2-Fc/FH_19-20_. Data were analysed using the Biacore S200 Evaluation Software 1.0. Responses from blank reference flow cells were subtracted from ligand-immobilised flow cells and all data were double-referenced (buffer inject subtracted).

### Flow Cytometric Analysis of B Cell Activation

Human peripheral blood mononuclear cells (PBMC) were isolated from leukocyte cone blood collected from healthy volunteers (NHS Blood and Transplant Service), using density-gradient centrifugation in LeucosepTM tubes (Greiner-Bio-One). PBMCs were frozen and stored in liquid nitrogen in accordance with UCB Biopharma UK HTA License Number 12,504. Frozen PBMCs were thawed and diluted into cold RPMI 1640 medium (Gibco) supplemented with 10% (v/v) foetal bovine serum, 1% (v/v) penicillin-streptomycin (Sigma Aldrich) and 1% (v/v) GlutaMAX (Gibco). For experiments on isolated B cells, B cells were purified from PBMCs by negative selection using the Miltenyi Biotec Human B Cell Isolation Kit II as per the manufacturer’s instructions. Following centrifugation, cells were counted, assessed for viability, which typically exceeded 90% for PBMCs and 80% for B cells, and re-suspended to the desired density in ambient medium.

PBMCs or B cells were then seeded onto sterile V-bottom plates at a density of 150,000 or 40,000 cells/well respectively and allowed to recover at 37°C with 5% CO_2_ for 1h. Monomeric C3d^17A^ or chemically-linked dimeric C3d^17C^ were serially diluted in media and added to the cells in duplicate to give final concentrations ranging from 2 µM to 0.1 nM (based on the molecular weight of monomeric C3d^17A^ for both constructs in order to control for the number of binding sites). Following a 30 min incubation period with the C3d constructs, additions of either goat F(ab’)_2_ anti-human IgM LE/AF (Southern Biotech) at a final concentration of 10 µg mL^-1^ or media were made and the cells incubated for a further 18h at 37°C with 5% CO_2_.

After a period of cooling on ice, the cells were stained for 1h with the LIVE/DEAD™ fixable near-infrared dead cell stain (1:1000 dilution, Invitrogen) along with the following labelled antibodies diluted in an ice-cold staining buffer (PBS supplemented with 1% BSA, 2mM EDTA and 0.05% NaN_3_): PerCP-Cy™5.5 mouse anti-human CD19 (1:40 dilution, Clone HIB19, BD Pharmingen) (for PBMC samples only), FITC mouse anti-human CD40 (1:20 dilution, Clone 5C3, BD Pharmingen), Brilliant Violet 421™ mouse anti-human CD69 (1:40 dilution, Clone FN50, BioLegend), PE mouse anti-human CD71 (1:20 dilution, Clone M-A712, BD Pharmingen) and APC mouse anti-human CD86 (1:20 dilution, Clone 2331, BD Pharmingen). The cells were subsequently washed and analysed on an Intellicyt^®^ iQue Screener PLUS flow cytometer. The gating strategy applied for live, singlet CD19^+^ B cells and activation markers can be found in **Supplementary Figures S12** and **S13**, respectively. Antibody capture beads were used for compensation. Data were expressed as mean values from at least 2 replicates ± standard deviation from the mean and depicted as scatter plots with curves fitted using a four-parameter variable slope non-linear regression model in GraphPad Prism (version 8.4.1). The geometric mean fluorescence intensity for the monomer and dimer were compared at both 0.1 nM and 2000 nM C3d concentration using an analysis of variance on the log transformed fluorescence fitting donor as a fixed effect. The downregulation of CD40 expression is expressed as a percentage reduction in fluorescence of the dimer compared to the monomer in the anti-IgM stimulated cells.

### Ca^2+^ Influx Experiments

Intracellular Ca^2+^ measurements using flow cytometry were performed as described previously (39–41). Briefly, isolated C57BL/6 mouse splenocytes maintained at 37°C were Indo 1-AM loaded, stained with a rat anti-mouse CD45R/B220-APC antibody (Clone RA3-6B2, BD Pharmingen) and analysed on a BD LSR II flow cytometer (BD Biosciences) at RT. 4 or 10 µg of monomeric C3d^17A^ or chemically-linked dimeric C3d^17C^ were added to the cells 30s after data acquisition. After 90s, cells were stimulated with a suboptimal concentration of pre-mixed complexes composed of 0.056 µg mL^-1^ biotinylated F(ab’)_2_ goat anti-mouse IgM (µ-chain specific) (Jackson ImmunoResearch), ~3 µg mL^-1^ C3dg-biotin (produced in house) and ~1.3 µg mL^-1^ streptavidin (aIgM-b/C3dg-b/ST). Experiments were run for 10 min and intracellular Ca^2+^ influx of gated B220^+^ cells was analysed using the FlowJo^®^ software (FlowJo LLC, BD).

## Results

To elucidate the importance of dimeric human C3 breakdown fragments, we investigated the formation of disulphide-linked dimers of C3b and C3d through limited trypsin proteolysis of C3 and subsequently analysed the structural characteristics of dimeric C3d using X-ray crystallography. SPR was used to compare the binding kinetics and avidity of these dimers and monomeric C3d to CR2 and FH_19-20_ and C3d-induced changes in the activation state of B cells were explored using flow cytometric analyses.

### Cleavage of C3 Results in the Spontaneous Formation of Disulphide-Linked C3b Dimers

**Figure 1:**
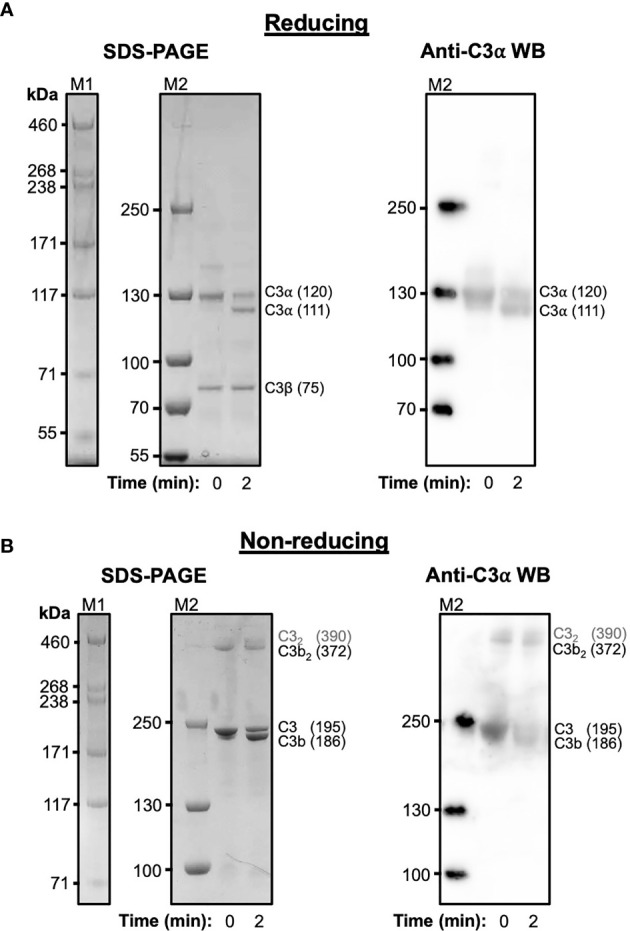
Cleavage of C3 results in the spontaneous formation of disulphide-linked C3b dimers. **(A)** Reducing Tris-Acetate SDS-PAGE (left panel) and anti-C3 α-chain western blot (right panel) analyses of serum-derived human C3 subjected to mild trypsin proteolysis at t=0 and t=2 minutes. Indicated are the intact and cleaved C3 α-chains (120 and 111 kDa, respectively) and C3 β-chain (75 kDa). Anti-C3 α-chain western blot analysis confirms the positions of the intact and cleaved C3α chains (right panel). **(B)** Non-reducing Tris-Acetate SDS-PAGE (left panel) and anti-C3 α-chain western blot (right panel) analyses of human C3 subjected to mild trypsin proteolysis at t=0 and t=2 minutes. Indicated are intact and cleaved C3 (195 and 186 kDa, respectively) as well as disulphide-linked C3b dimers (C3b_2_) and a faint band suggestive of a dimeric form of C3 (highlighted as C3_2_ in grey font). Anti-C3 α-chain western blot analysis confirms the positions of intact C3 and monomeric and disulphide-linked dimeric C3b (right panel). Molecular weight markers shown are HiMark (M1) and PageRuler Plus (M2). Raw SDS-PAGE gel and western blot images can be found in **Supplementary Figure S1**.

C3 purified from human serum was cleaved with trypsin under mild proteolysis conditions and subsequently analysed using SDS-PAGE and anti-C3 α-chain western blotting (**Figure 1**). Over 50% of C3 is cleaved to C3b following digestion with trypsin after 2 minutes (**Figure 1A**) and a significant fraction of this C3b, visualised under non-reducing conditions, spontaneously forms disulphide-bonded dimers (**Figure 1B**). These dimers form instantly and remain stable for at least 2 hours (**Supplementary Figure S1**). In addition to the C3b dimers, a weak higher molecular weight band suggestive of a dimeric form of C3 can also be observed at t=0. The absence of these dimers in samples treated with reducing agent indicates their formation is mediated by disulphide bonds. In a similar manner, recombinant wild-type human C3d, with its native thioester cysteine intact (C3d^17C^), also forms disulphide-linked dimers under non-reducing conditions (**Supplementary Figure S2**).

### Disulphide Linkage of the Thioester Cysteine Results in C3d Dimerisation

**Figure 2:**
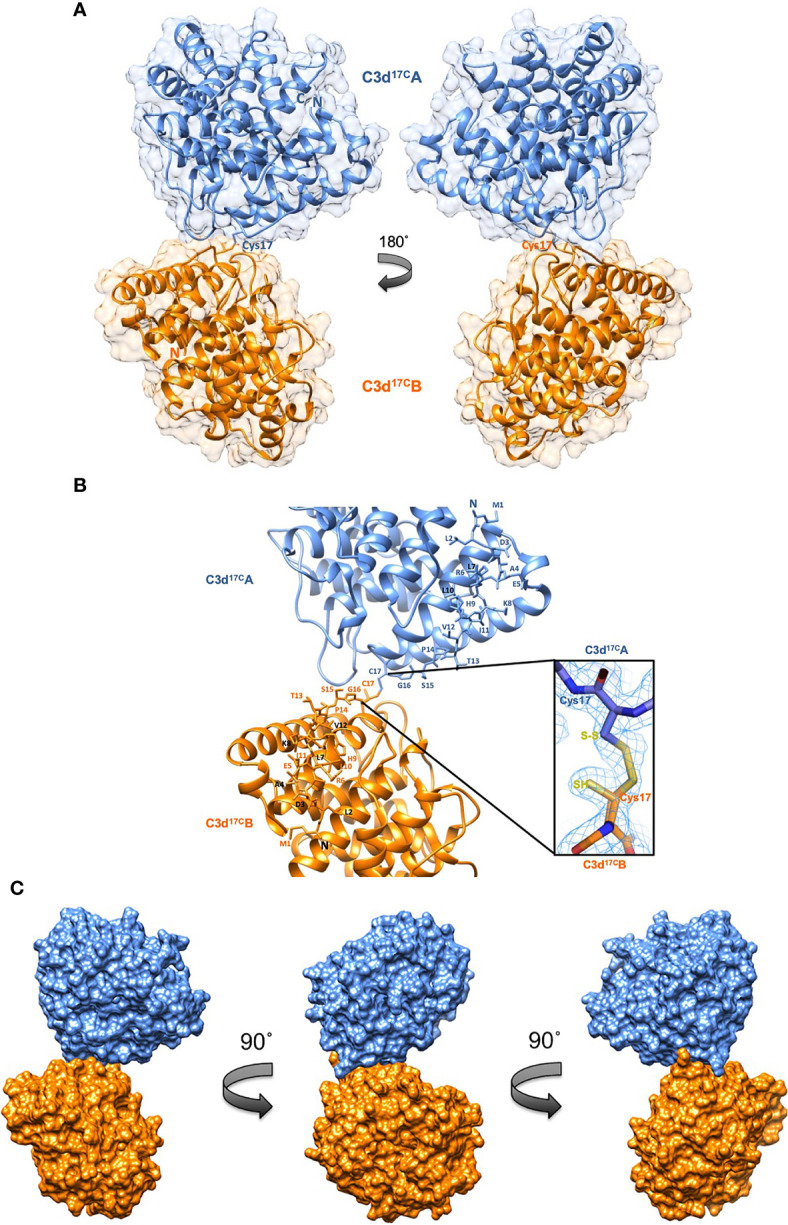
Structure of a disulphide-linked human C3d^17C^ dimer at 2.0 Å resolution. **(A)** The ribbon diagram shows disulphide linkage of the monomeric subunits at position Cys17 results in the formation of a dimer 92.37 Å in length with a 0.61 Å (chain A)/0.40 Å (chain B) main chain (M1-P294) RMSD relative to the structure of C3d^17A^ (PDB:1C3D). **(B)** Enlarged view of the C3d^17C^ dimer interface showing the side chains of helix α1 residues M1-C17. Inset: 2Fo-Fc electron density contoured at 1.0 σ of the partially broken C17-C17 interchain disulphide bond (2.07 Å) resulting from oxidation of one conformer of Chain B Cys17. **(C)** Solid molecular surface representation of the C3d^17C^ dimer in three different orientations rotated by 90° angles counter-clockwise. PDB accession code: 6RMT. See **Supplementary Tables S1, S2** for data collection and refinement statistics.

A crystal structure of wild-type human C3d, harbouring a cysteine at position 17/1010 (C3d numbering/intact pre-pro C3 numbering) (C3d^17C^), was obtained at 2.0 Å resolution (**Figure 2**, **Supplementary Tables S1, S2**). The structure clearly shows the formation of a dimer mediated by partial disulphide linkage of the thioester cysteine residues at position 17/1010 in both monomeric chains. This 17C-17C disulphide creates a link between the two C3d monomers at the C-terminus of helix α1, causing the convex molecular surfaces of the monomers to orient towards each other whilst simultaneously exposing their concave binding faces (**Figure 2A**). Closer examination of the C3d^17C^ dimer interaction surface (**Figure 2B**) confirms that the overall (α-α)_6_ barrel configuration of both monomers remains unchanged and comparable to previously-published structures of monomeric C3d (0.61 Å (chain A)/0.40 Å (chain B) main chain (M1-P294) RMSD relative to PDB: 1C3D). The 2Fo-Fc electron density map at the C3d^17C^ dimer interface shows that chain B residue 17C (**Figure 2B** inset) adopts a dual conformation with one conformer existing in an unpaired oxidized form, perhaps due to radiation damage. This indicates the disulphide bond linking the two C3d monomers can occur in a partially disconnected state which is consistent with results from size exclusion chromatography experiments suggesting C3d^17C^ exists in a an almost equivalent monomer-dimer ratio in solution (**Supplementary Figure S2**).

Superimposition of the ligand-binding domains of CR2 (SCR1-2), the α_M_I integrin domain of CR3 or SCRs 19-20 of FH on to the dimeric C3d^17C^ structure does not generate any molecular clashes (**Supplementary Figures S3A–D**). This important observation suggests dimerisation does not cause any interference in the formation of complexes between C3d or C3dg and their most physiologically-relevant binding partners. *Staphylococcus aureus* immune evasion proteins such as Efb-C, Ecb/Ehp and Sbi-IV are also predicted to bind the C3d^17C^ dimer without any hindrance as the concave surfaces of both monomers are exposed and accessible. Significantly, as CR2 and CR3 interact with the C3d^17C^ dimer *via* opposing surfaces, complement receptor crosslinking could play an important role in the function of C3d dimers (**Supplementary Figure S3C**). Moreover, the absence of steric hindrance following superimposition of the C3d^17C^ dimer onto the C3b TED domain (**Supplementary Figure S3E**), suggests dimerisation of C3b, as proposed previously (42, 43), could occur in a similar fashion to C3d without affecting the ability of C3b to interact with the complement regulators FH and FI.

### C3d Dimers Can Crosslink CR2 and FH_19-20_

As our structural analyses revealed the propensity of C3d^17C^ to dimerise, we next analysed the binding interactions of C3d dimers in comparison to monomeric C3d^17A^ using CR2 and FH_19-20_ as two important known binding partners. Given that our size exclusion chromatography experiments (**Supplementary Figure S2**) showed that monomeric and dimeric C3d exist in relatively equal amounts in solution we opted to create chemically stable dimers of C3d^17C^ conjugated at the 17C position *via* a bromine-based linear linker (N,N’-(propane-1,3-diyl) bis(2-bromoacetamide), see *Materials and Methods*; **Supplementary Figures S4–S6B**). The N,N’-(propane-1,3-diyl) bis(2-bromoacetamide) linker was used as this class of chemical compound has been shown to selectively crosslink cysteine residues located within close spatial proximity (44). Dimeric C3d^17C^ resulting from this chemical crosslinking reaction was subsequently validated using particle analysis (**Supplementary Figure S6C**), analytical ultracentrifugation (**Supplementary Figure S6D**) and mass spectrometry (**Supplementary Tables S3, S4** and **Supplementary Figure S6E**) and utilised in SPR spectroscopy studies to gain insights into its binding patterns and kinetics.

**Figure 3:**
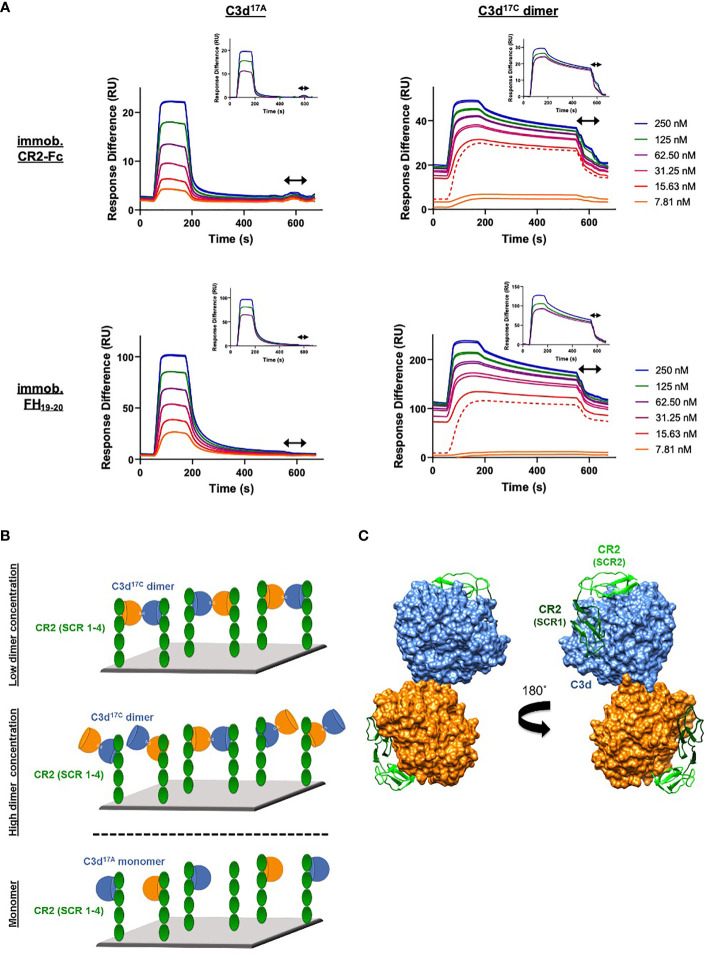
Dimeric C3d^17C^ crosslinks CR2 and FH_19-20_. **(A)** SPR sensorgrams showing serially-diluted concentrations of 250 nM monomeric C3d^17A^ (left) or dimeric C3d^17C^ (right) flowed in duplicate over flow cells of a CM5 sensor chip immobilised with CR2-Fc (top) or FH_19-20_ (bottom). The binding of C3d^17A^ to CR2-Fc and FH_19-20_ follows a conventional association-steady state-dissociation pattern while the binding of dimeric C3d^17C^ to the same ligands generates an unusual two-state binding interaction. At concentrations up to the first 15.63 nM replicate (dashed line) the binding patterns depict highly avid interactions suggestive of the formation of dimeric C3d^17C^-CR2-Fc and dimeric C3d^17C^-FH_19-20_ crosslinked complexes which are not fully eluted from the surface. Thus, the subsequent injection cycles commence at a higher baseline response where the high avidity binding sites for dimeric C3d^17C^ on CR2-Fc or FH_19-20_ remain saturated. This causes the binding mode to switch to less favourable, readily-disrupted interactions suggestive of the formation of 1:1 complexes, although some crosslinked complexes persist. Inset: baseline-adjusted sensorgrams showing the less favourable 1:1 complexes (1 C3d^17C^ dimer: 1 CR2-Fc or FH_19-20_ molecule) which form at higher concentrations of dimeric C3d^17C^ (62.5-250 nM) and are readily eluted from the surface. Arrows depict the regeneration period. See **Supplementary Figure S7** for further details and **Supplementary Figure S8** for results from an additional two independent experiments. **(B)** Schematic model depicting the proposed mechanistic basis behind dimeric C3d^17C^-mediated crosslinking of surface-associated CR2 (SCR 1-4). At low concentrations, C3d^17C^ dimers crosslink two surface-associated CR2 (SCR 1-4) molecules *via* highly avid interactions involving the acidic residue-lined concave face of C3d and the basic amino acid-rich SCRs 1 and 2 of CR2 (top). Once a critical threshold concentration has been surpassed, the increase in dimeric C3d^17C^ molecules relative to available CR2 binding sites outcompetes the second binding site on C3d^17C^ dimers and favours the formation of 1:1 complexes (middle). Unlike C3d^17C^ dimers, monomeric C3d^17A^ lacks the ability to crosslink CR2 and is restricted to the formation of 1:1 complexes (bottom). **(C)** Superposition of SCR1-2 of CR2 (PDB accession code: 3OED) onto its binding sites on the C3d^17C^ dimer demonstrating how dimeric C3d^17C^ could crosslink CR2, as indicated by the SPR data gathered, at a structural level.

In contrast to monomeric C3d^17A^, which displays a conventional association-steady state-dissociation binding pattern when flowed over surface-immobilised CR2-Fc and FH_19-20_ (**Figure 3A** left), the binding of dimeric C3d^17C^ to the same ligands was found to be noticeably distinct and suggestive of a two-state binding interaction (**Figure 3A** right). At low concentrations up to the first replicate of 15.63 nM (dashed line), the highly avid interactions with negligible dissociation indicate a bivalent binding mode whereby the C3d^17C^ dimer crosslinks two CR2-Fc or two FH_19-20_ molecules. During the first injection of 15.63 nM C3d^17C^ dimer, 25 RU of material binds to the surface and 10 RU remain avidly bound to the surface after regeneration. While at the second 15.63 nM injection, 18 RU of material binds to the surface and only 2RU remains avidly bound at the end (**Supplementary Figure S7**). In both cases an equivalent amount of material is eluted during regeneration, indicating the first injection likely saturates the highly avid binding sites. As the surface cannot be fully regenerated of these high avidity complexes, the subsequent cycle commences at a higher baseline response. At this point and higher concentrations, the high avidity binding sites for dimeric C3d^17C^ on CR2-Fc or FH_19-20_ remain saturated causing the binding mode to switch to less favourable/avid readily-disrupted interactions suggestive of 1:1 binding between the C3d^17C^ dimer and CR2-Fc or FH_19-20_ although some cross-linked C3d^17C^ dimer-CR2-Fc and C3d^17C^ dimer- FH_19-20_ complexes persist (1-2 RU of material remaining bound to the surface after regeneration). At the highest three concentrations of dimeric C3d^17C^ (62.5-250 nM), the less favourable 1:1 interactions (between 1 C3d^17C^ dimer: 1 CR2-Fc or FH_19-20_ molecule) which are readily eluted from the surface dominate (**Figure 3A** right inset). Consistent with these results, the unusual binding patterns observed here were also evident in a further two independent experiments (**Supplementary Figure S8**) and cannot be attributed to higher order species of analyte or ligand as biophysical techniques showed the dimeric C3d^17C^ and FH_19-20_ preparations used did not contain aggregates (**Supplementary Figures S6C, D** and **S9**). A model illustrating the binding events described here is presented in **Figure 3B** (for CR2-Fc) and the superposition displayed in **Figure 3C** illustrates the feasibility of CR2 crosslinking by dimeric C3d^17C^ at a structural level.

### Dimeric C3d Is a More Potent Modulator of B Cell Activation Than Monomeric C3d

Following on from our SPR studies which indicated dimeric C3d may have the capacity to crosslink CR2, our next aim was to analyse the effects of dimeric compared to monomeric C3d on B cell activation. Flow cytometry was employed to examine changes in the expression of four surface-associated B cell activation markers (CD40, CD69, CD71 and CD86) resulting from stimulation of isolated human B cells with monomeric C3d^17A^ or chemically-linked dimeric C3d^17C^ alone or in the presence of BCR-crosslinking anti-IgM F(ab^’^)_2_. As shown in **Supplementary Figure S10**, although agonism of the BCR by anti-IgM significantly enhances expression of all the activation markers (except CD40), neither monomeric C3d^17A^ nor dimeric C3d^17C^ appears to influence the activation of isolated B cells in an appreciable manner, as measured by the markers examined.

**Figure 4:**
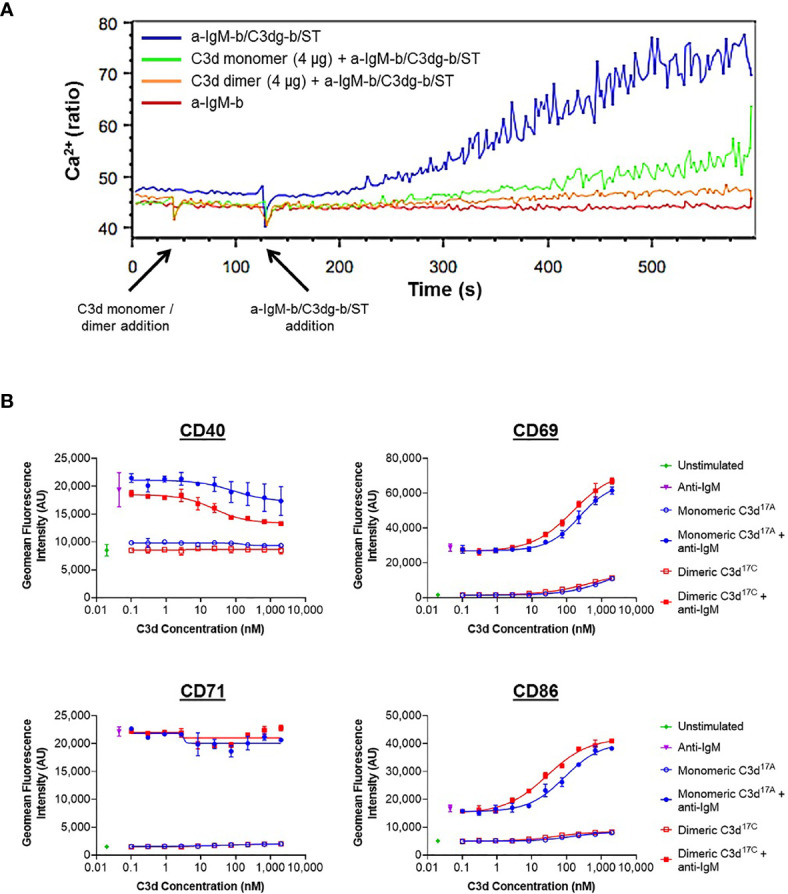
Monomeric C3d^17A^ and to a greater extent dimeric C3d^17C^ alter the activation state of murine **(A)** and human **(B)** B cell populations. **(A)** Ca^2+^ influx experiment showing incubation with 4 µg C3d^17A^ monomer or C3d^17C^ dimer (30 s) 90 seconds prior to the addition of BCR/CR2-crosslinking complexes (a-IgM-b/C3dg-b/ST) (120 s) significantly retards and reduces Ca^2+^ influx in CD45R/B220-gated Indo 1-AM-loaded C57BL/6 mouse splenocytes with a more pronounced blocking effect apparent with dimeric C3d^17C^. 10 µg of either form of C3d completely eliminates Ca^2+^ influx (**Supplementary Figure S11**) suggesting the observed blocking effect is concentration dependent and likely a result of CR2 sequestration by monomeric C3d^17A^/dimeric C3d^17C^ reducing the proportion of CR2 available for crosslinking with the BCR. BCR/CR2-crosslinking complexes were composed of a suboptimal dose (0.056 µg mL^-1^) of biotinylated F(ab’)_2_ goat anti‐mouse IgM (a-IgM-b), C3dg-biotin (C3dg-b) and streptavidin (ST). The C3d^17A^ monomer/C3d^17C^ dimer-mediated blocking of Ca^2+^ influx was not evident when higher, more optimal concentrations of a-IgM-b/ST were used or when all the reaction components were added simultaneously. **(B)** Flow cytometric analysis of CD19^+^ B cells stimulated with monomeric C3d^17A^ or dimeric C3d^17C^ in the presence or absence of BCR-crosslinking anti-IgM F(ab^’^)_2_ (10 µg mL^-1^) reveals C3d-induced changes in the expression of surface-associated B cell activation markers. While no C3d-mediated changes in CD71 expression are evident, at higher concentrations (≥ 3 nM) both monomeric C3d^17A^ and dimeric C3d^17C^ appear to downregulate CD40, with a more pronounced reduction in expression in the presence of dimeric C3d^17C^. Conversely, in the presence of anti-IgM, both monomeric C3d^17A^ and to a greater extent dimeric C3d^17C^ synergistically upregulate CD69 and CD86 although at concentrations ≥ 10 nM both forms of C3d are also capable of enhancing expression of these activation markers in the absence of anti-IgM. Data are of PBMC B cell populations from a representative donor and displayed as mean values (n=2) ± standard deviation from the mean with curves fitted using a non-linear regression model. Results from an additional two donors can be found in **Supplementary Figure S14**.

A more general approach, using Ca^2+^ influx as a measure of B cell activation was therefore taken next. Here, incubation of B220^+^ mouse splenocytes with monomeric C3d^17A^ or dimeric C3d^17C^ prior to stimulation with a suboptimal dose of a biotinylated- anti-IgM/C3dg-biotin/streptavidin (a-IgM-b/C3dg-b/ST) BCR/CR2-crosslinking complex was found to inhibit BCR/CR2-mediated Ca^2+^ influx in a concentration-dependent manner (**Figure 4A** and **Supplementary Figure S11**). The observed blocking effect was more pronounced following treatment with dimeric C3d^17C^, particularly at the lower concentration of 4 µg (**Figure 4A**), and for both constructs is only evident when C3d is added ahead of the a-IgM-b/C3dg-b/ST complex and when a suboptimal dose of anti-IgM (i.e. unable to trigger Ca^2+^ influx in the absence of CR2 engagement) within the crosslinking complex is used. Thus, the perceived inhibition of Ca^2+^ influx and hence B cell activation is likely to result from sequestration of CR2 by monomeric C3d^17A^, and to a greater extent, due to avidity and possibly *via* CR2-CR2 crosslinking as suggested by our SPR experiments, dimeric C3d^17C^, reducing the proportion of CR2 available for crosslinking with the BCR.

In order to further investigate C3d-mediated changes in the activation state of B cells within mixed populations of cells, as would occur *in vivo*, flow cytometry was utilised to explore differences in the expression of CD40, CD69, CD71 and CD86 on CD19^+^ cells within donor human PBMC samples (see **Supplementary Figures S12** and **S13** for gating strategy applied). In contrast to the results gathered from isolated human B cells (**Supplementary Figure S10**), a clear dose-dependent relationship between C3d and B cell activation was observed in these experiments indicating other mononuclear cell types may be involved in B cell-responsiveness to free C3d, as measured by expression of the markers analysed (**Figure 4B**). At concentrations ≥ 10 nM, both monomeric C3d^17A^ and chemically-linked dimeric C3d^17C^ are able to enhance expression of the early B cell activation markers CD69 and CD86 even in the absence of BCR engagement by anti-IgM. In concert with anti-IgM although both forms of C3d synergistically upregulate expression of these markers in a concentration-dependent manner, dimeric C3d^17C^ is found to be approximately three-fold more effective at enhancing activation than monomeric C3d^17A^ (47nM vs 139 nM (CD69) and 18 nM vs 59 nM (CD86) geometric mean EC50s), perhaps through more avid interactions with CR2.

Interestingly, in contrast to CD69 and CD86, both monomeric C3d^17A^ and dimeric C3d^17C^ appear to downregulate CD40, particularly in the presence of anti-IgM, with a more pronounced reduction in expression evident in the presence of dimeric C3d^17C^. Differently still, despite achieving a substantial increase in expression in the presence of anti-IgM in the experimental time period, CD71 does not appear to be influenced by either form of C3d. Importantly, the differential marker-specific trends observed are consistent across cells gathered from all three donors analysed (data from donors 2 and 3 can be found in **Supplementary Figure S14**) suggesting free C3d (unattached to an antigen) may regulate B cell activation in a selective manner and that dimeric C3d may have more potent modulatory roles than C3d monomers.

## Discussion

Pre-treatment of C3b with sulfhydryl-alkylating agents and routine use of a recombinant thioester cysteine deletion construct (C17A) in the past has prohibited the structural and functional analysis of disulphide-linked dimers of C3 fragments that can form following activation in the fluid phase. Concurrent with previous findings (25), in this study we provide evidence showing trypsin-mediated cleavage of C3 results in the spontaneous formation of a significant fraction of disulphide-linked C3b dimers (**Figure 1**). Interestingly, our results additionally suggest the formation of a dimeric form of C3. This dimeric fraction could conceivably involve the hydrolysed form of C3 (C3(H_2_O)) in which the exposed thioester cysteine sulphydryl renders it prone to the formation of disulphide-linked dimers as observed with C3b (25) as well as the related thioester-containing complement protein fragments C4Ab and C4Bb (45).

Furthermore, here we verify that C3 breakdown product C3d, with its native thioester cysteine intact (C3d^17C^), forms disulphide-linked dimers in an analogous fashion to C3b and in the first X-ray crystal structure of a human C3d dimer we confirm that dimerisation is mediated by disulphide linkage of the thioester cysteine residues at position 17/1010 (C3d numbering/intact pre-pro C3 numbering) (**Figure 2**) in a manner that would also permit the analogous dimerisation of C3b (**Supplementary Figure S3E**). Importantly, this dimer retains the ability of C3d to bind SCR domains 1-2 of CR2, the α_M_I integrin domain of CR3 and SCR domains 19-20 of FH (**Supplementary Figures S3A–D**).

In order to complement our structural studies, we next analysed the binding of a stable chemically-linked C3d^17C^ dimer (**Supplementary Figures S5, S6**) to CR2 (SCR1-4) and FH_19-20_ using SPR. Here dimeric C3d^17C^ showed higher avidity binding to both of the interacting partners examined, and in contrast to monomeric C3d, was found to crosslink surface-associated CR2 as well as FH_19-20_ (**Figure 3**, **Supplementary Figures S7** and **S8**). This crosslinking by disulphide-linked C3d^17C^ dimers cannot be explained by the formation of higher order aggregates of dimeric C3d^17C^ (**Supplementary Figures S6C, D**) or FH_19-20_ (**Supplementary Figure S9**) and is a finding that has not been observed previously but could indicate a potential physiologically-relevant role of these dimers. Future investigations will elucidate whether dimeric C3d^17C^ can crosslink its other receptor, CR3, or a combination of CR2 and CR3, as suggested by our structural superpositions (**Supplementary Figures S3B, C**).

Finally, we investigated the effects of dimeric compared to monomeric C3d on the activation state of primary human and murine B cells using flow cytometry and Ca^2+^ influx experiments. In contrast to results from resting tonsillar B cells (46), when assayed in isolation, we found B cells purified from human PBMCs appeared to be unresponsive to both forms of free C3d (**Supplementary Figure S10**). However, both monomeric C3d^17A^, and to a greater extent dimeric C3d^17C^, inhibited BCR/CR2-mediated Ca^2+^ influx in B220^+^ murine splenocytes when added prior to stimulation with a BCR/CR2-crosslinking complex (**Figure 4A** and **Supplementary Figure S11**). Further to previous reports using biotinylated C3dg (with a C17A mutation) in the presence of streptavidin (39, 46), these results suggest that pre-ligation of CR2 by naturally-occurring fluid phase C3d(g) dimers could inhibit BCR/CR2 crosslinking-mediated Ca^2+^ responses in B cells by sequestering the CR2/CD19/CD81 receptor complex from the BCR with higher avidity than C3d monomers.

Both dimeric and monomeric C3d were also found to induce changes in the expression of B cell activation markers on human CD19^+^ cells within PBMC samples (**Figure 4B** and **Supplementary Figure S14**). Specifically, in the presence of anti-IgM, both monomeric C3d^17A^ and to a three-fold greater extent dimeric C3d^17C^, synergistically upregulated CD69 and CD86 which is consistent with previous reports showing independent ligation of CR2 and the BCR (i.e. without crosslinking) by simultaneous stimulation with biotinylated-C3dg/streptavidin complexes and anti-IgM can augment B cell activation (47). Our results, however, additionally show that dimeric C3d^17C^ is more efficient at augmenting CR2/BCR-dependent activation and that BCR engagement may not be necessary for upregulation of certain activation markers as at higher concentrations both forms of C3d were also capable of enhancing expression of the early activation markers CD69 and CD86 in the absence of anti-IgM. Although these findings in human PBMCs differ from a recent report suggesting C3d inhibits the BCR-induced expression of CD69 on isolated tonsillar B cells (46), they are likely more representative of *in vivo* conditions, where interactions between different cell types and associated factors occur continuously.

In contrast to CD69 and CD86, both monomeric and dimeric C3d appeared to downregulate CD40, with a more pronounced reduction in expression in the presence of dimeric C3d^17C^. CD40 is involved in the regulation of several B cell processes including germinal centre reactions (48), isotype switching (49) and somatic hypermutation (50) and has also been shown to prevent B cells from becoming anergic (51). Although further investigations are required to explain the C3d-mediated downregulation of CD40 expression on B cells observed in our study, it is possible that C3d stimulation of CR2 or CR3 expressed on other PBMC cell types [e.g. T cells (52–55) and natural killer cells (56)] induces the production of higher levels of soluble CD40L that drive internalisation of CD40 or prevent efficient staining by occluding the receptor. Alternatively, the known binding of CD40L to CR3 (57) could be outcompeted by CR3 interactions with C3d, particularly in its dimeric form, elevating the levels of soluble CD40L available for binding CD40. Further experiments investigating the effects of free monomeric and dimeric C3d on IgG titre and hence B-cell differentiation or antibody class switching following activation of PBMCs with T cell supernatants or co-culture with IL-2 or CD40L-producing feeder cells will help to understand this process further.

Nonetheless, the preliminary data presented here suggest fluid-phase C3d(g), particularly in its dimeric form, could alter the activation of B cells and may direct them towards an anergic state. Although further verification is required, this proposed role would be logical in terms of helping to limit the involvement of complement in the generation of humoral immune responses in the absence of a threat and is consistent with reports of CR2 ligation being involved in the anergy of autoreactive B cells (47, 58, 59). Thus, in the future, C3d(g) dimers could have implications for the development of novel therapies for autoimmune diseases, for example through their effects on CD40/CD40L interactions, particularly as downregulation of CD40 was shown to be a beneficial outcome of Rituximab treatment of systemic lupus erythematosus (SLE) patients (60) and CD40/CD40L levels have been linked to anti-DNA autoantibody titres in lupus patients (61) and mouse models (62). By extension, these newly-uncovered functions of C3d could also offer a possible explanation as to why humoral immune responses are inhibited, rather than enhanced, by certain vaccine constructs composed of antigens linked to linear repeats of C3d placed in close proximity to each other (47, 63).

On the whole, our cell experiments not only suggest free fluid-phase C3d(g) (unattached to a surface) may regulate B cell activation in a selective manner but also that there are clear functional differences between monomeric and dimeric C3d with the latter being a more potent modulator of the activation state of B cells as a consequence of high avidity receptor interactions or through receptor crosslinking. In addition, they indicate other PBMC cell types play an important role in the responsiveness of B cells, in terms of the activation markers analysed, to C3d, perhaps through the provision of sensitising or synergising co-stimulatory molecules or *via* CR2-CR2 or CR2-CR3 crosslinking between cells. Although these preliminary experiments have brought to light some of the potentially physiologically-relevant functions of fluid-phase C3d(g) dimers, further investigations probing the molecular mechanisms underlying these roles are warranted. In a wider context, it would also be interesting to explore possible connections between the levels of C3d(g) dimers, their distribution in the body and pathological conditions associated with uncontrolled C3 activation, such as C3 glomerulopathy, as we surmise local upregulation of fluid phase C3d(g) concentrations is likely to enhance C3d(g) dimerisation.

In summary, in this study we reaffirm the spontaneous formation of disulphide-linked C3b dimers following cleavage of C3 and present the first structure of a fluid phase disulphide-linked human C3d dimer. Through accompanying functional analyses we show that these dimers could have physiologically-relevant roles in crosslinking CR2 and selectively modulating B cell activation, possibly to trigger tolerogenic pathways. Overall, our findings shed light on a fundamental aspect of complement biology that is often overlooked and could have the potential to inform the design of novel therapeutics for immune system disorders in the future.

## Data Availability Statement

The datasets presented in this study can be found in online repositories. The names of the repository/repositories and accession number(s) can be found in the article/**Supplementary Material**.

## Author Contributions

JE, KM, AM, BG, and AAW designed the experiments. CB performed preliminary structural studies. AAW performed the crystallisation and circular dichroism experiments. SC and AAW reprocessed the crystallography data and refined the structures. RD purified C3, guided by ML, and carried out the trypsin proteolysis, molecular modelling and structural analyses. TH produced and purified the FH constructs. AGW, BA, and RM synthesised and characterised the linker and conducted initial linkage experiments. BG and AAW performed the SPR experiments under the guidance of KM and with helpful discussions from CH who also analysed the data. LK completed the Ca^2+^ influx experiments. The B cell activation flow cytometry experiments were performed by AM and KW with the assistance of IM. JE and AAW wrote the manuscript with valuable contributions from all the authors. All authors contributed to the article and approved the submitted version.

## Funding

This research was supported by the Biotechnology and Biological Sciences Research Council Follow On Fund BB/N022165/1. AAW was sponsored by a PhD studentship granted by Raoul and Catherine Hughes and the University of Bath Alumni Fund. RD was supported by a Medical Research Council GW4 Doctoral Training Partnership. BG and KM were funded by a Northern Counties Kidney Research Grant and Alexion Pharmaceuticals funded TH’s PhD studentship *via* Complement UK. KM also recognises the support from Kidney Research UK grant (RP-006-20270301). CH was funded by Newcastle University. 

## Conflict of Interest

KM is a consultant for and receives funding or renumeration from Gemini Therapeutics Ltd., Freeline Therapeutics, MPM Capital and Catalyst Biosciences.

CH has recently received consultancy or SAB payments from Freeline Therapeutics, Q32 Bio Inc., Roche, GlaxoSmithKline and Gyroscope Therapeutics and has received research funding from Ra Pharmaceuticals; all funds were donated to Newcastle University. TH is funded by Alexion Pharmaceuticals. Authors AM, KW, IM, and AL are or were employed by UCB-Pharma and may hold shares and/or stock options.

The remaining authors declare that the research was conducted in the absence of any commercial or financial relationships that could be construed as a potential conflict of interest.

## Publisher’s Note

All claims expressed in this article are solely those of the authors and do not necessarily represent those of their affiliated organizations, or those of the publisher, the editors and the reviewers. Any product that may be evaluated in this article, or claim that may be made by its manufacturer, is not guaranteed or endorsed by the publisher.
