# Supplementary material for: Insights Into the Structure-Function Relationships of Dimeric C3d Fragments
Source: Front Immunol. 2021 Aug 9;12:714055. doi: 10.3389/fimmu.2021.714055 (PMC8381054; doi:10.3389/fimmu.2021.714055)
Supplement: Supplementary file 1 [file Presentation_1.pptx]

## Slide 1
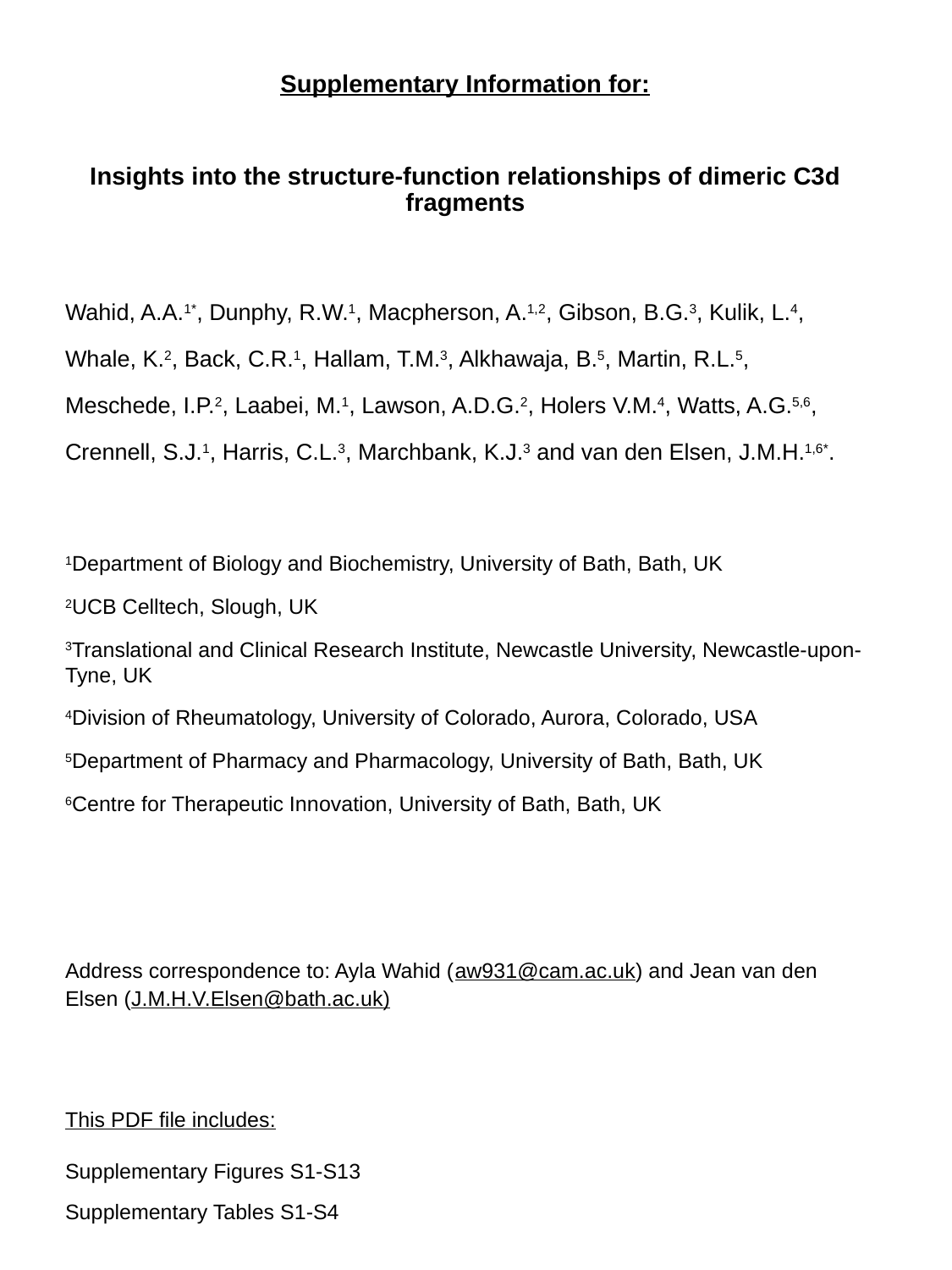

Supplementary Information for:
Insights into the structure-function relationships of dimeric C3d fragments
Wahid, A.A.1*, Dunphy, R.W.1, Macpherson, A.1,2, Gibson, B.G.3, Kulik, L.4, Whale, K.2, Back, C.R.1, Hallam, T.M.3, Alkhawaja, B.5, Martin, R.L.5, Meschede, I.P.2, Laabei, M.1, Lawson, A.D.G.2, Holers V.M.4, Watts, A.G.5,6, Crennell, S.J.1, Harris, C.L.3, Marchbank, K.J.3 and van den Elsen, J.M.H.1,6*.
1Department of Biology and Biochemistry, University of Bath, Bath, UK
2UCB Celltech, Slough, UK
3Translational and Clinical Research Institute, Newcastle University, Newcastle-upon-Tyne, UK
4Division of Rheumatology, University of Colorado, Aurora, Colorado, USA
5Department of Pharmacy and Pharmacology, University of Bath, Bath, UK
6Centre for Therapeutic Innovation, University of Bath, Bath, UK
Address correspondence to: Ayla Wahid (aw931@cam.ac.uk) and Jean van den Elsen (J.M.H.V.Elsen@bath.ac.uk)
This PDF file includes:
Supplementary Figures S1-S13
Supplementary Tables S1-S4

## Slide 2
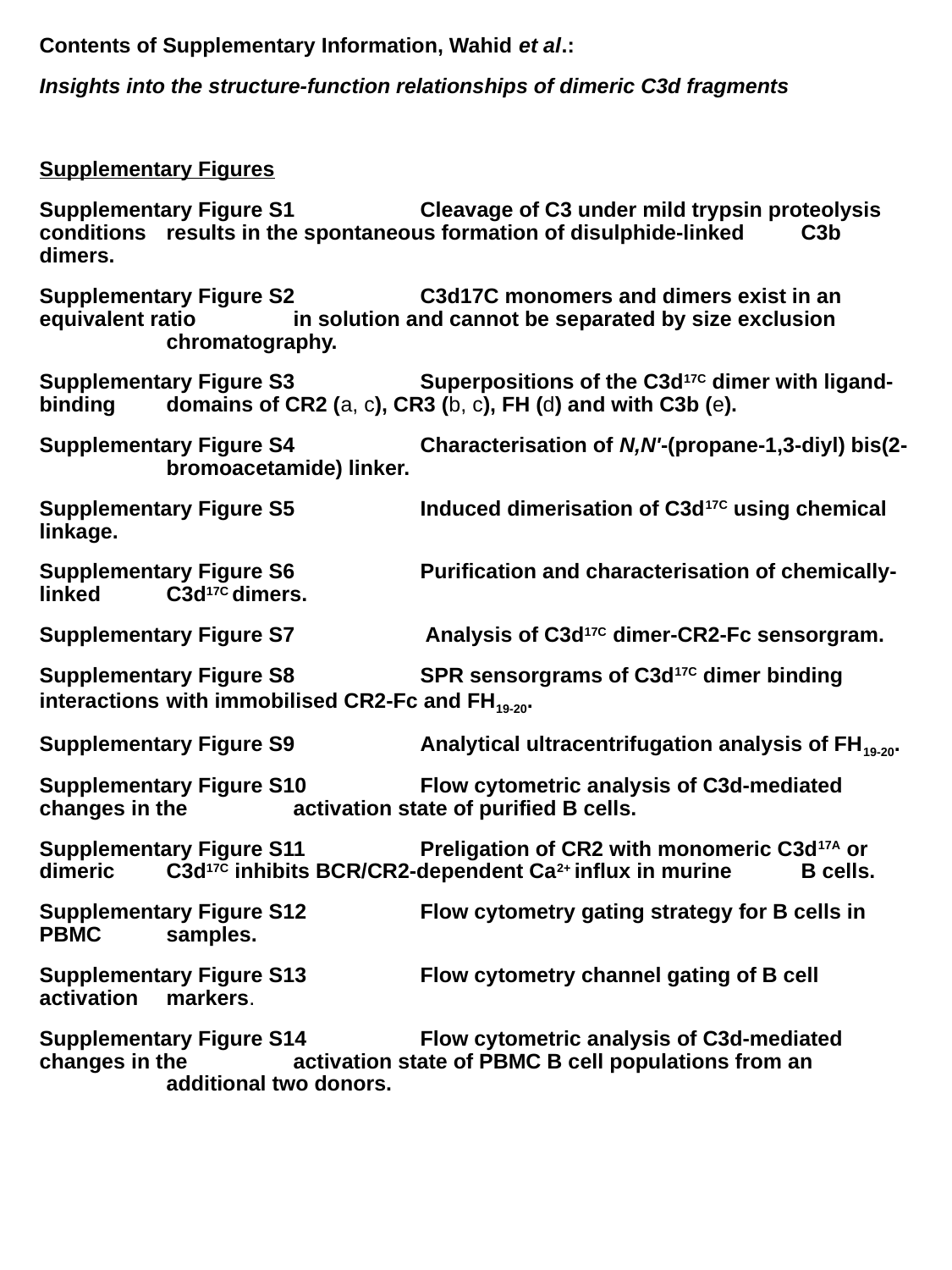

Contents of Supplementary Information, Wahid et al.:
Insights into the structure-function relationships of dimeric C3d fragments
Supplementary Figures
Supplementary Figure S1	Cleavage of C3 under mild trypsin proteolysis conditions 	results in the spontaneous formation of disulphide-linked 	C3b dimers.
Supplementary Figure S2	C3d17C monomers and dimers exist in an equivalent ratio 	in solution and cannot be separated by size exclusion 	chromatography.
Supplementary Figure S3 	Superpositions of the C3d17C dimer with ligand-binding 	domains of CR2 (a, c), CR3 (b, c), FH (d) and with C3b (e).
Supplementary Figure S4 	Characterisation of N,N'-(propane-1,3-diyl) bis(2-	bromoacetamide) linker.
Supplementary Figure S5	Induced dimerisation of C3d17C using chemical linkage.
Supplementary Figure S6	Purification and characterisation of chemically-linked 	C3d17C dimers.
Supplementary Figure S7	 Analysis of C3d17C dimer-CR2-Fc sensorgram.
Supplementary Figure S8	SPR sensorgrams of C3d17C dimer binding interactions 	with immobilised CR2-Fc and FH19-20.
Supplementary Figure S9 	Analytical ultracentrifugation analysis of FH19-20.
Supplementary Figure S10 	Flow cytometric analysis of C3d-mediated changes in the 	activation state of purified B cells.
Supplementary Figure S11	Preligation of CR2 with monomeric C3d17A or dimeric 	C3d17C inhibits BCR/CR2-dependent Ca2+ influx in murine 	B cells.
Supplementary Figure S12	Flow cytometry gating strategy for B cells in PBMC 	samples.
Supplementary Figure S13	Flow cytometry channel gating of B cell activation 	markers.
Supplementary Figure S14	Flow cytometric analysis of C3d-mediated changes in the 	activation state of PBMC B cell populations from an 	additional two donors.

## Slide 3
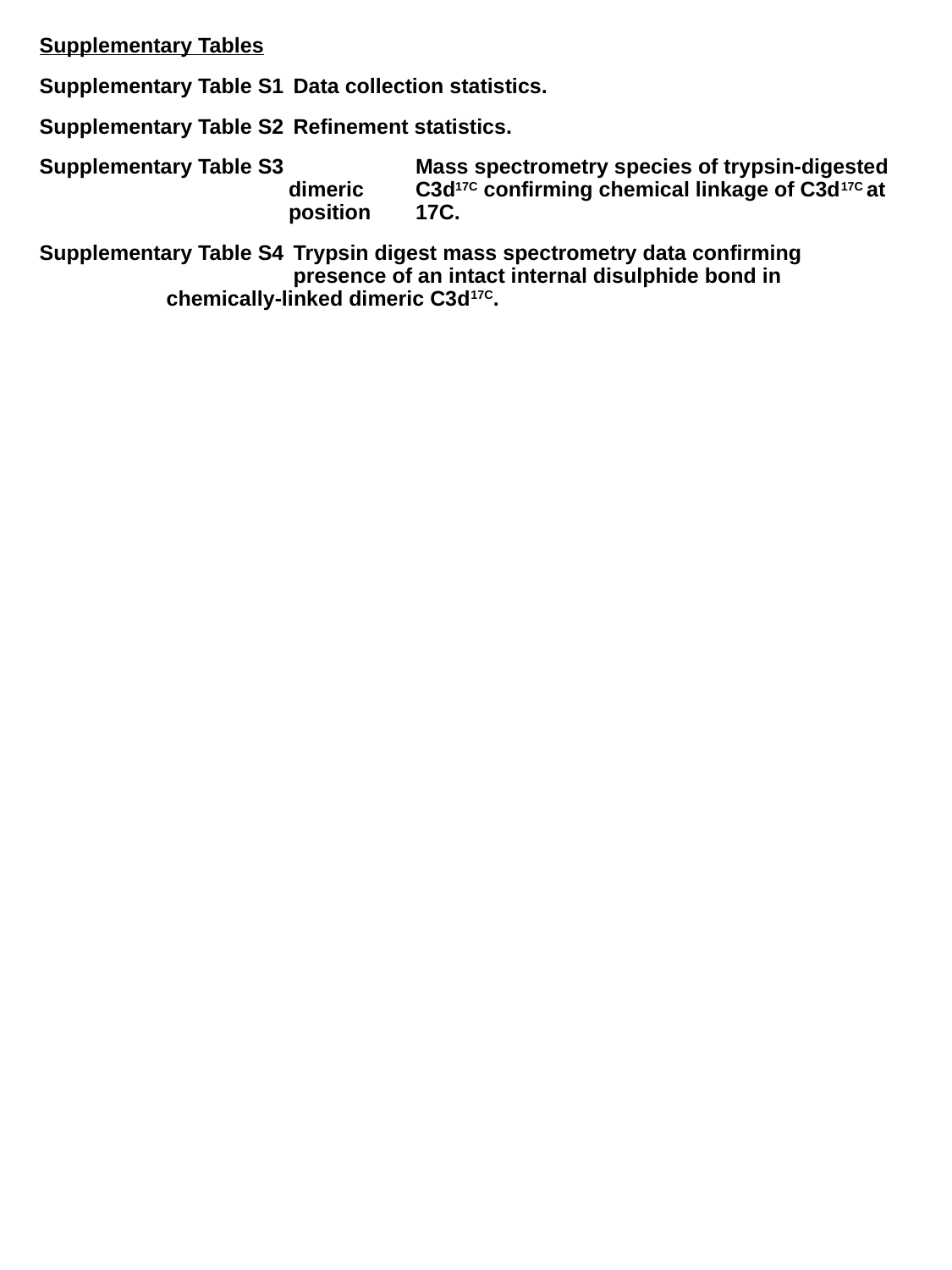

Supplementary Tables
Supplementary Table S1	Data collection statistics.
Supplementary Table S2 	Refinement statistics.
Supplementary Table S3 	Mass spectrometry species of trypsin-digested dimeric 	C3d17C confirming chemical linkage of C3d17C at position 	17C.
Supplementary Table S4 	Trypsin digest mass spectrometry data confirming 		presence of an intact internal disulphide bond in 		chemically-linked dimeric C3d17C.

## Slide 4
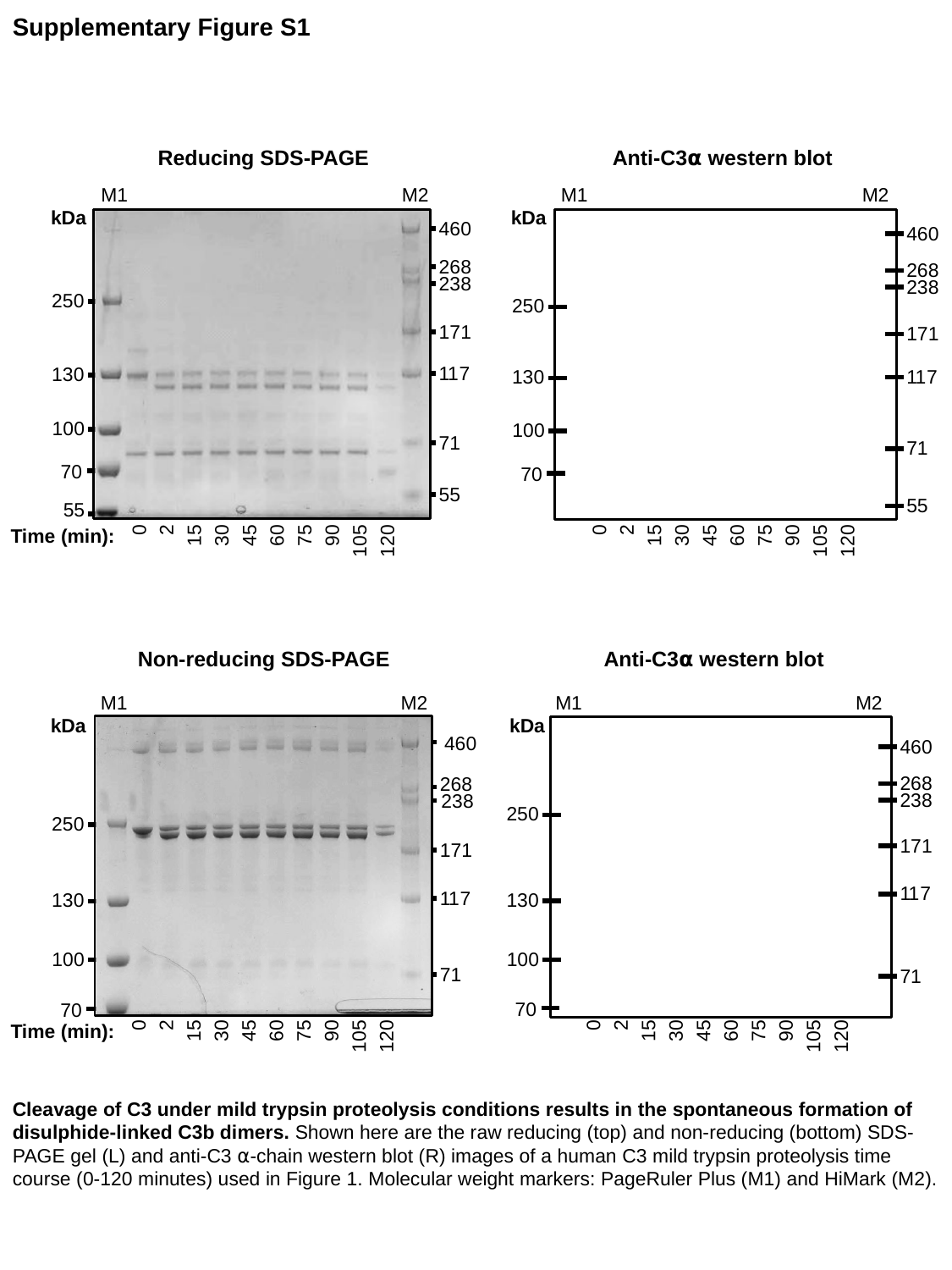

Supplementary Figure S1
Reducing SDS-PAGE
Anti-C3⍺ western blot
Non-reducing SDS-PAGE
Anti-C3⍺ western blot
Cleavage of C3 under mild trypsin proteolysis conditions results in the spontaneous formation of disulphide-linked C3b dimers. Shown here are the raw reducing (top) and non-reducing (bottom) SDS-PAGE gel (L) and anti-C3 ⍺-chain western blot (R) images of a human C3 mild trypsin proteolysis time course (0-120 minutes) used in Figure 1. Molecular weight markers: PageRuler Plus (M1) and HiMark (M2).

## Slide 5
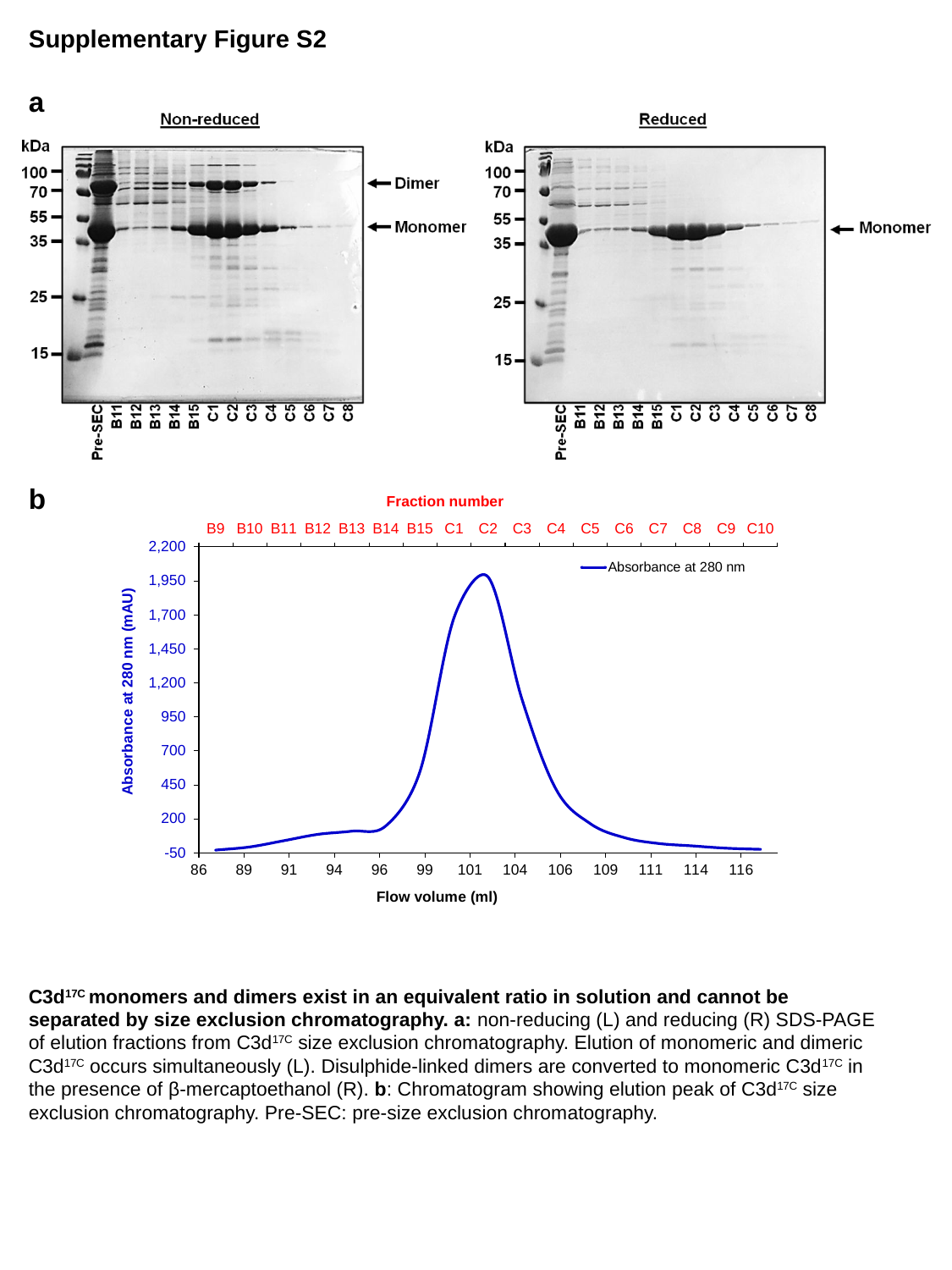

Supplementary Figure S2
a
b
C3d17C monomers and dimers exist in an equivalent ratio in solution and cannot be separated by size exclusion chromatography. a: non-reducing (L) and reducing (R) SDS-PAGE of elution fractions from C3d17C size exclusion chromatography. Elution of monomeric and dimeric C3d17C occurs simultaneously (L). Disulphide-linked dimers are converted to monomeric C3d17C in the presence of β-mercaptoethanol (R). b: Chromatogram showing elution peak of C3d17C size exclusion chromatography. Pre-SEC: pre-size exclusion chromatography.

## Slide 6
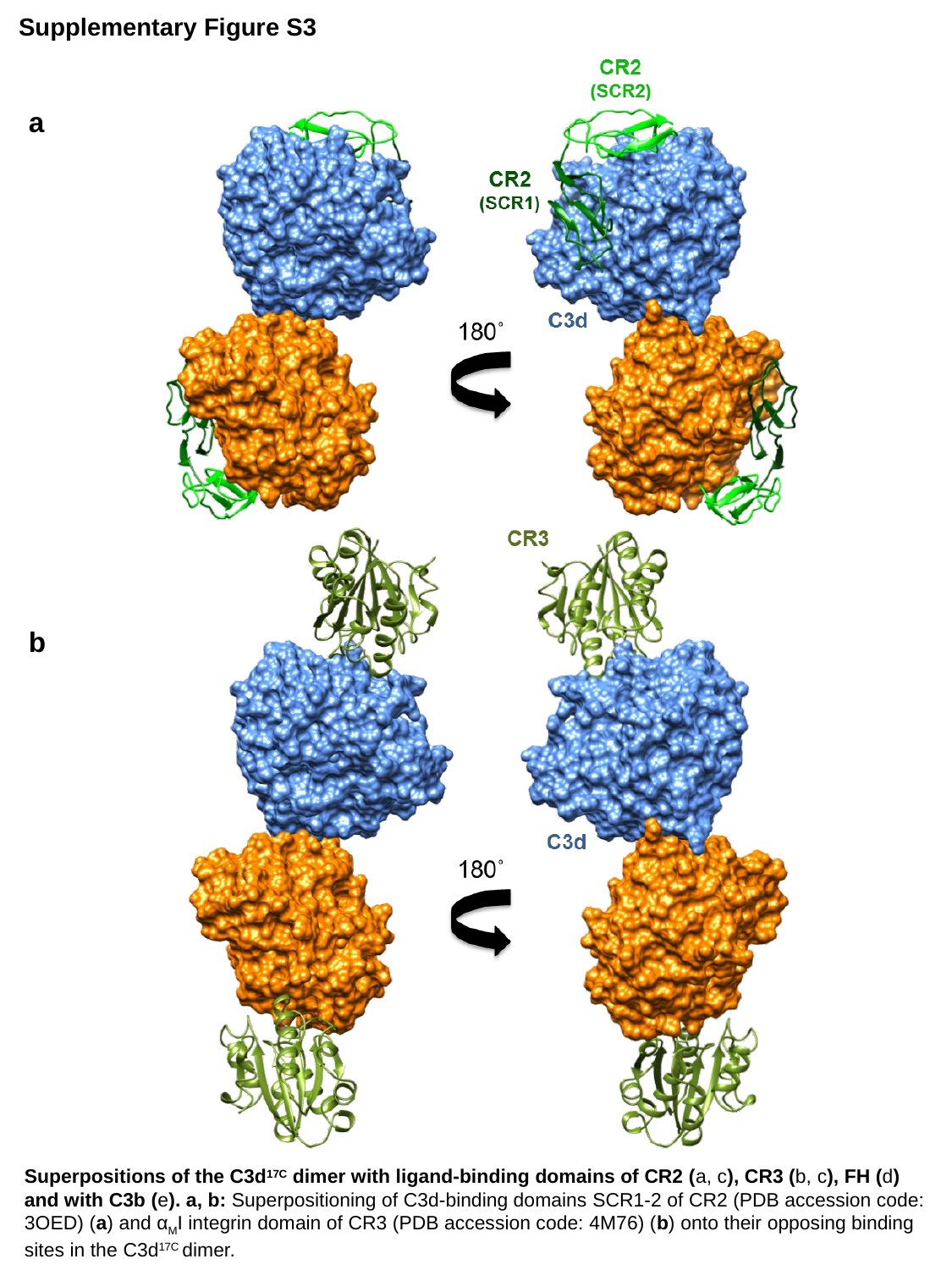

Supplementary Figure S3
a
b
Superpositions of the C3d17C dimer with ligand-binding domains of CR2 (a, c), CR3 (b, c), FH (d) and with C3b (e). a, b: Superpositioning of C3d-binding domains SCR1-2 of CR2 (PDB accession code: 3OED) (a) and αMI integrin domain of CR3 (PDB accession code: 4M76) (b) onto their opposing binding sites in the C3d17C dimer.

## Slide 7
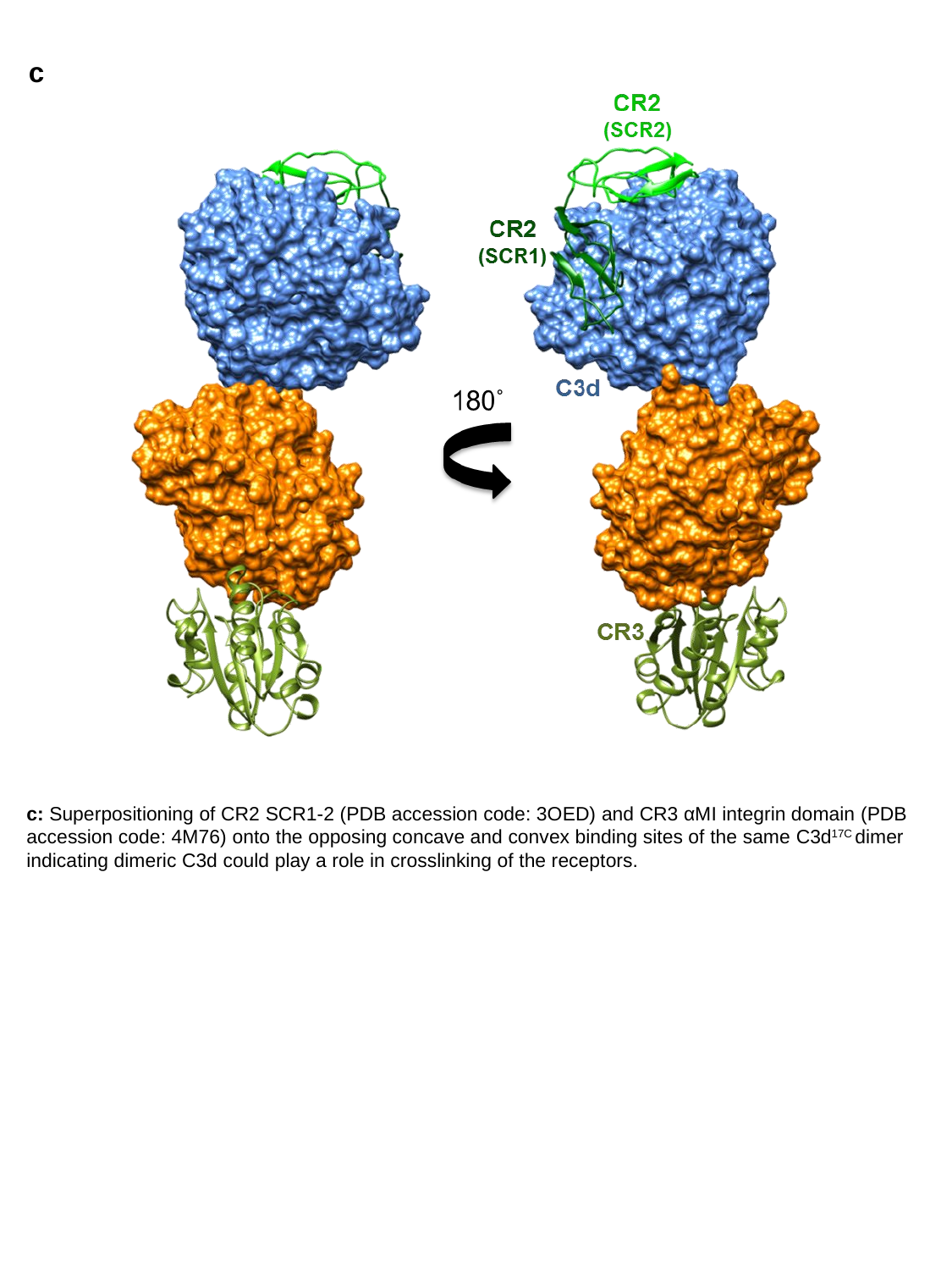

c
c: Superpositioning of CR2 SCR1-2 (PDB accession code: 3OED) and CR3 αMI integrin domain (PDB accession code: 4M76) onto the opposing concave and convex binding sites of the same C3d17C dimer indicating dimeric C3d could play a role in crosslinking of the receptors.

## Slide 8
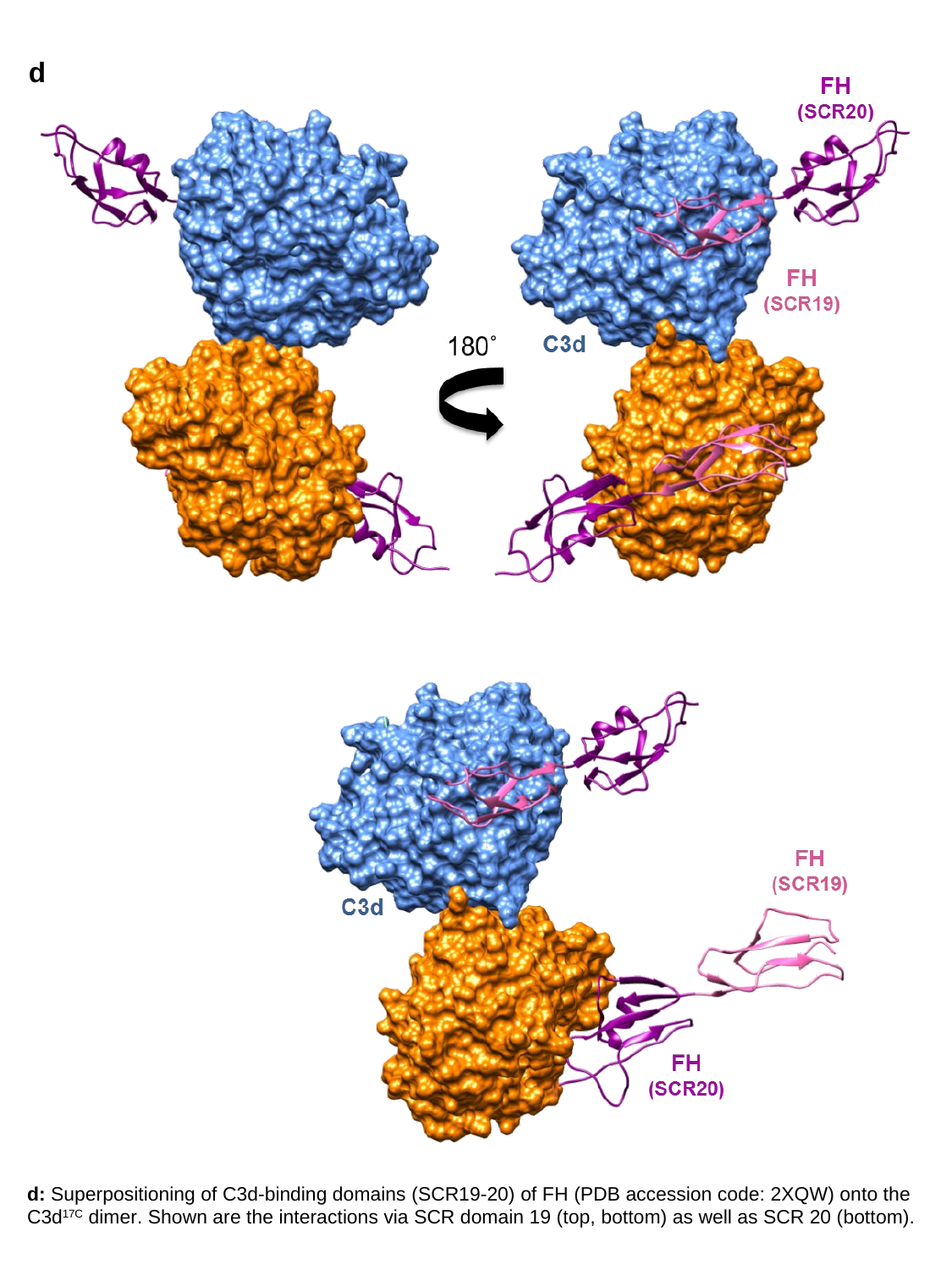

d
d: Superpositioning of C3d-binding domains (SCR19-20) of FH (PDB accession code: 2XQW) onto the C3d17C dimer. Shown are the interactions via SCR domain 19 (top, bottom) as well as SCR 20 (bottom).

## Slide 9
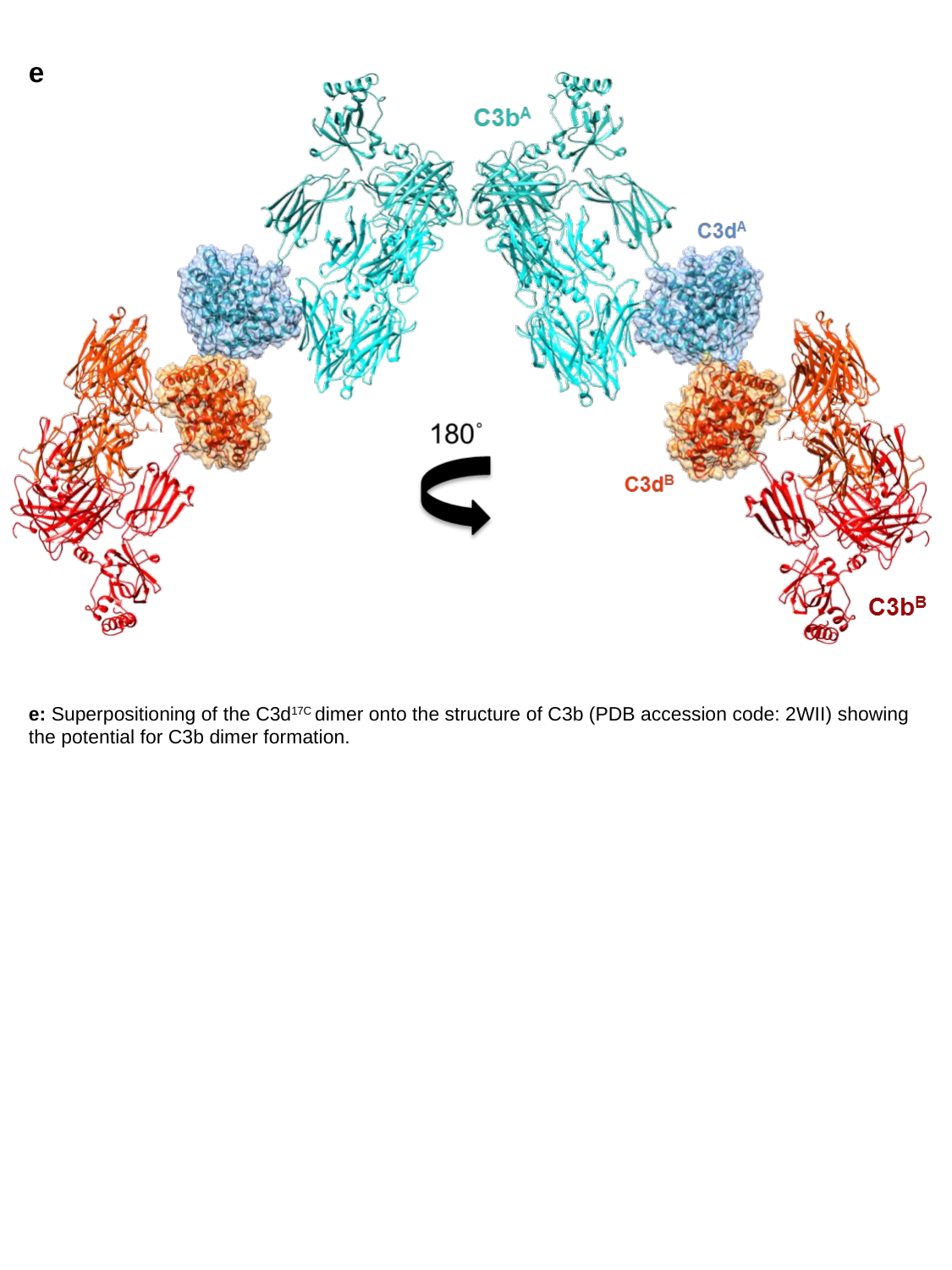

e
e: Superpositioning of the C3d17C dimer onto the structure of C3b (PDB accession code: 2WII) showing the potential for C3b dimer formation.

## Slide 10
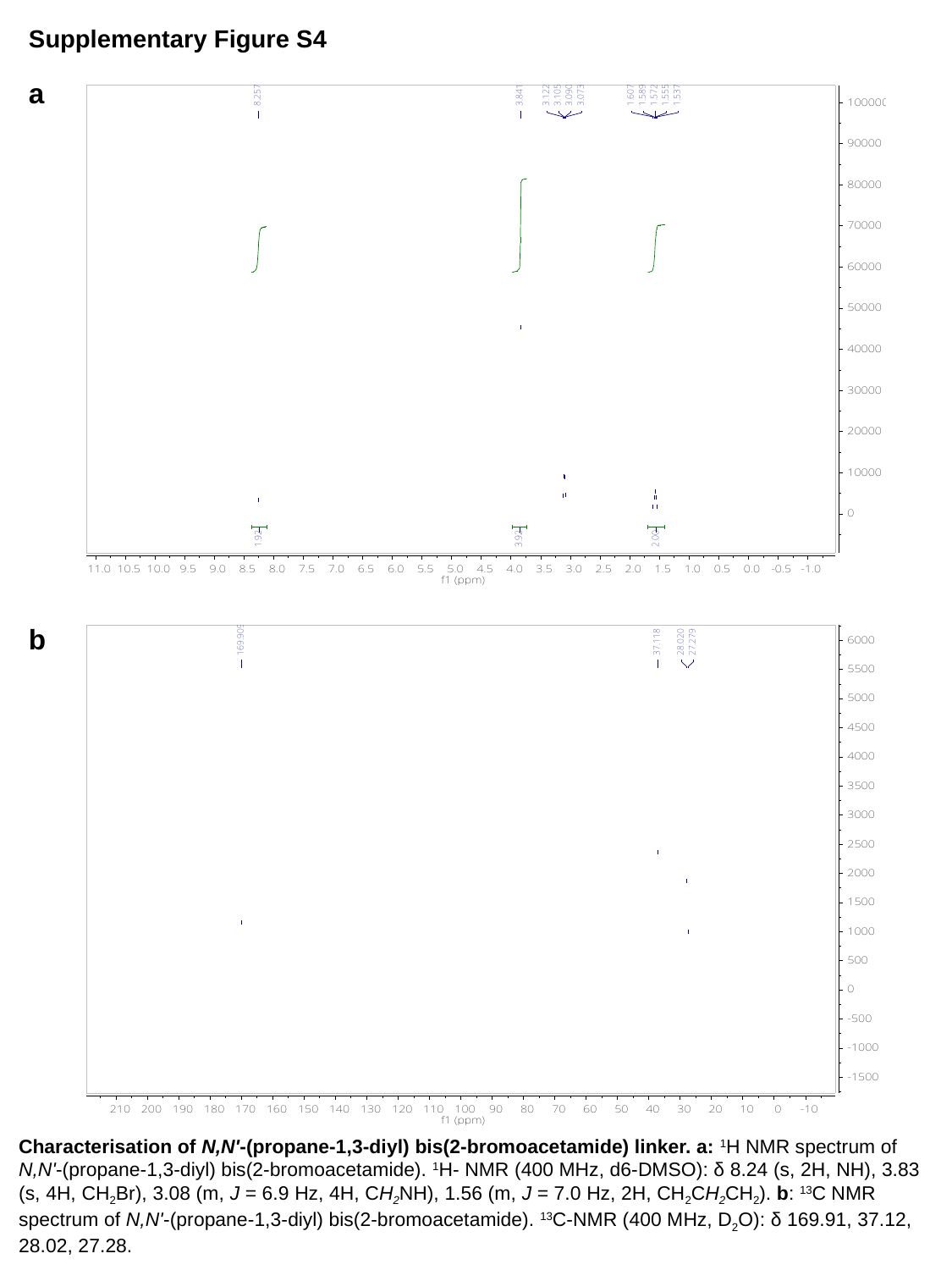

Supplementary Figure S4
a
b
Characterisation of N,N'-(propane-1,3-diyl) bis(2-bromoacetamide) linker. a: 1H NMR spectrum of N,N'-(propane-1,3-diyl) bis(2-bromoacetamide). 1H- NMR (400 MHz, d6-DMSO): δ 8.24 (s, 2H, NH), 3.83 (s, 4H, CH2Br), 3.08 (m, J = 6.9 Hz, 4H, CH2NH), 1.56 (m, J = 7.0 Hz, 2H, CH2CH2CH2). b: 13C NMR spectrum of N,N'-(propane-1,3-diyl) bis(2-bromoacetamide). 13C-NMR (400 MHz, D2O): δ 169.91, 37.12, 28.02, 27.28.

## Slide 11
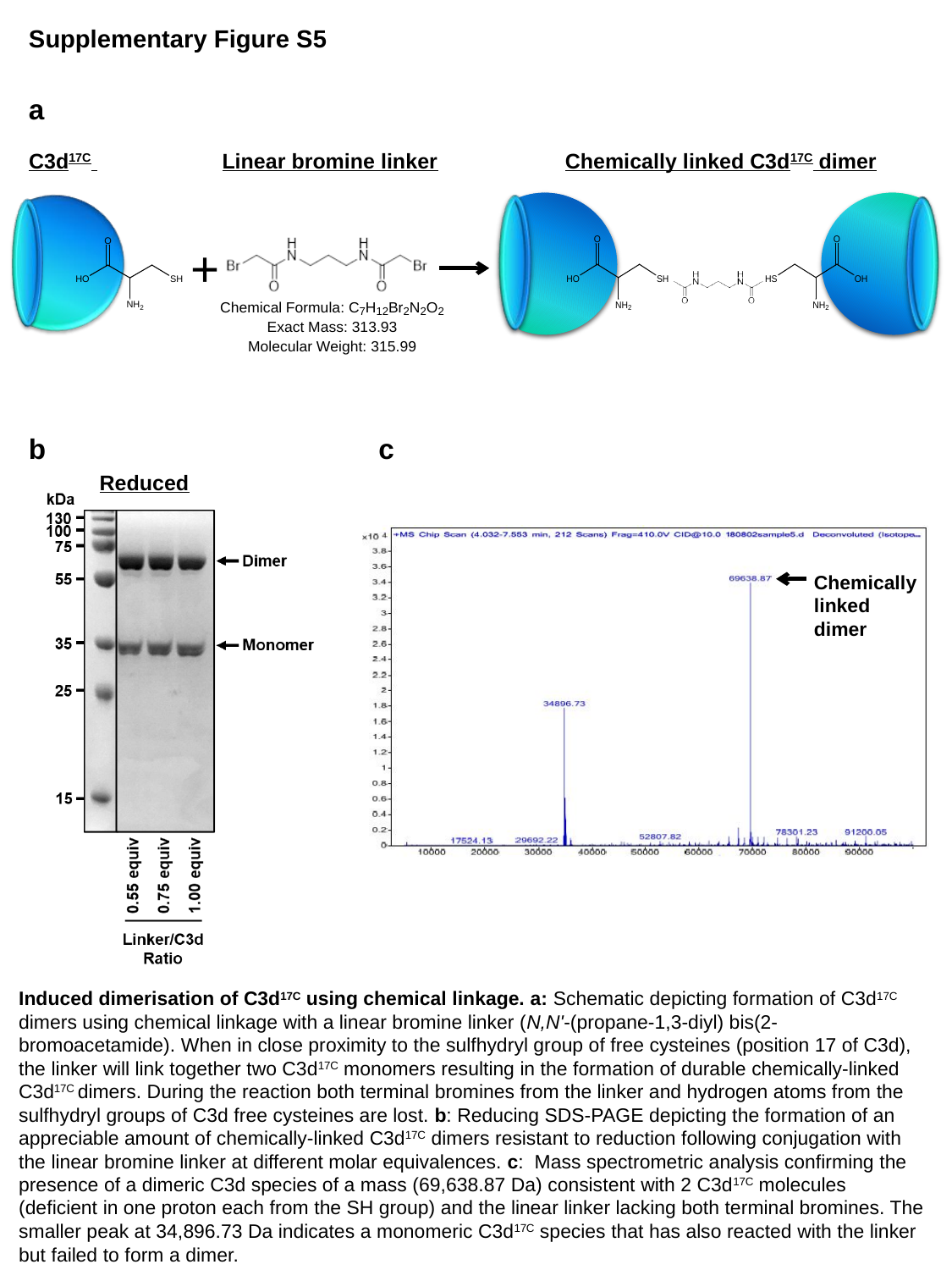

Supplementary Figure S5
a
C3d17C
Linear bromine linker
Chemically linked C3d17C dimer
+
b
c
Reduced
Chemically
linked dimer
Induced dimerisation of C3d17C using chemical linkage. a: Schematic depicting formation of C3d17C dimers using chemical linkage with a linear bromine linker (N,N'-(propane-1,3-diyl) bis(2-bromoacetamide). When in close proximity to the sulfhydryl group of free cysteines (position 17 of C3d), the linker will link together two C3d17C monomers resulting in the formation of durable chemically-linked C3d17C dimers. During the reaction both terminal bromines from the linker and hydrogen atoms from the sulfhydryl groups of C3d free cysteines are lost. b: Reducing SDS-PAGE depicting the formation of an appreciable amount of chemically-linked C3d17C dimers resistant to reduction following conjugation with the linear bromine linker at different molar equivalences. c: Mass spectrometric analysis confirming the presence of a dimeric C3d species of a mass (69,638.87 Da) consistent with 2 C3d17C molecules (deficient in one proton each from the SH group) and the linear linker lacking both terminal bromines. The smaller peak at 34,896.73 Da indicates a monomeric C3d17C species that has also reacted with the linker but failed to form a dimer.

## Slide 12
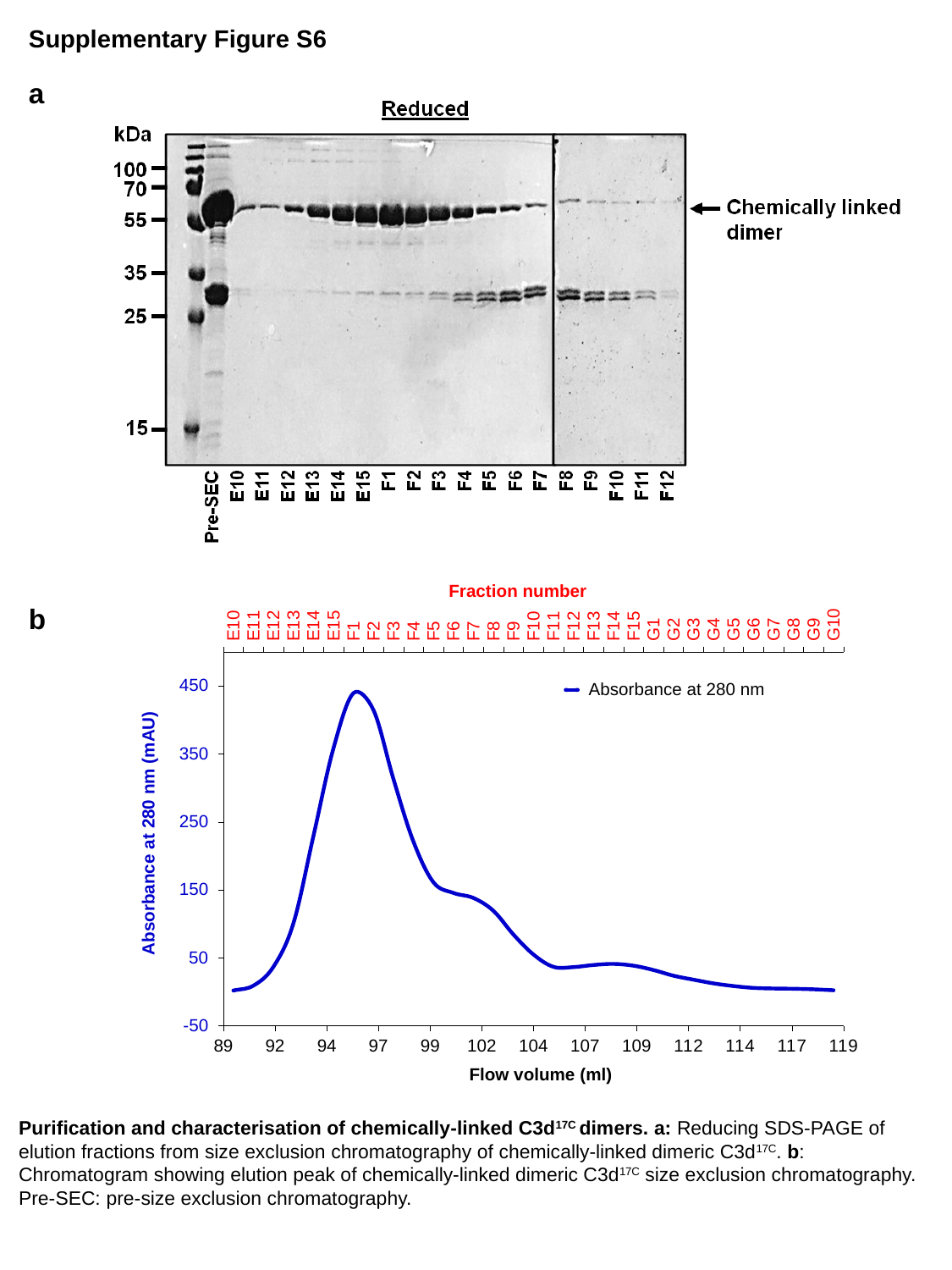

Supplementary Figure S6
a
b
Purification and characterisation of chemically-linked C3d17C dimers. a: Reducing SDS-PAGE of elution fractions from size exclusion chromatography of chemically-linked dimeric C3d17C. b: Chromatogram showing elution peak of chemically-linked dimeric C3d17C size exclusion chromatography. Pre-SEC: pre-size exclusion chromatography.

## Slide 13
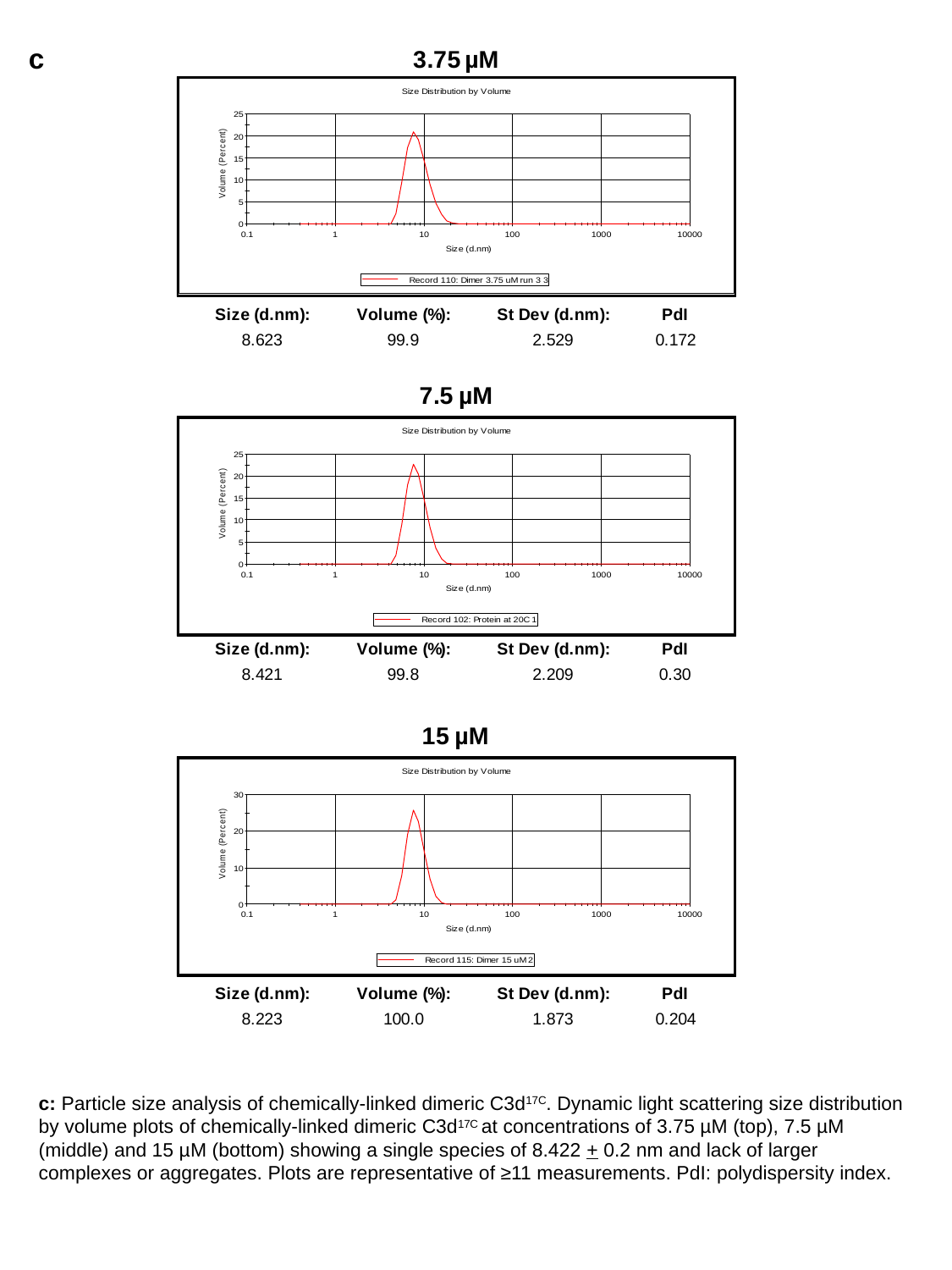

c
c: Particle size analysis of chemically-linked dimeric C3d17C. Dynamic light scattering size distribution by volume plots of chemically-linked dimeric C3d17C at concentrations of 3.75 µM (top), 7.5 µM (middle) and 15 µM (bottom) showing a single species of 8.422 + 0.2 nm and lack of larger complexes or aggregates. Plots are representative of ≥11 measurements. PdI: polydispersity index.

## Slide 14
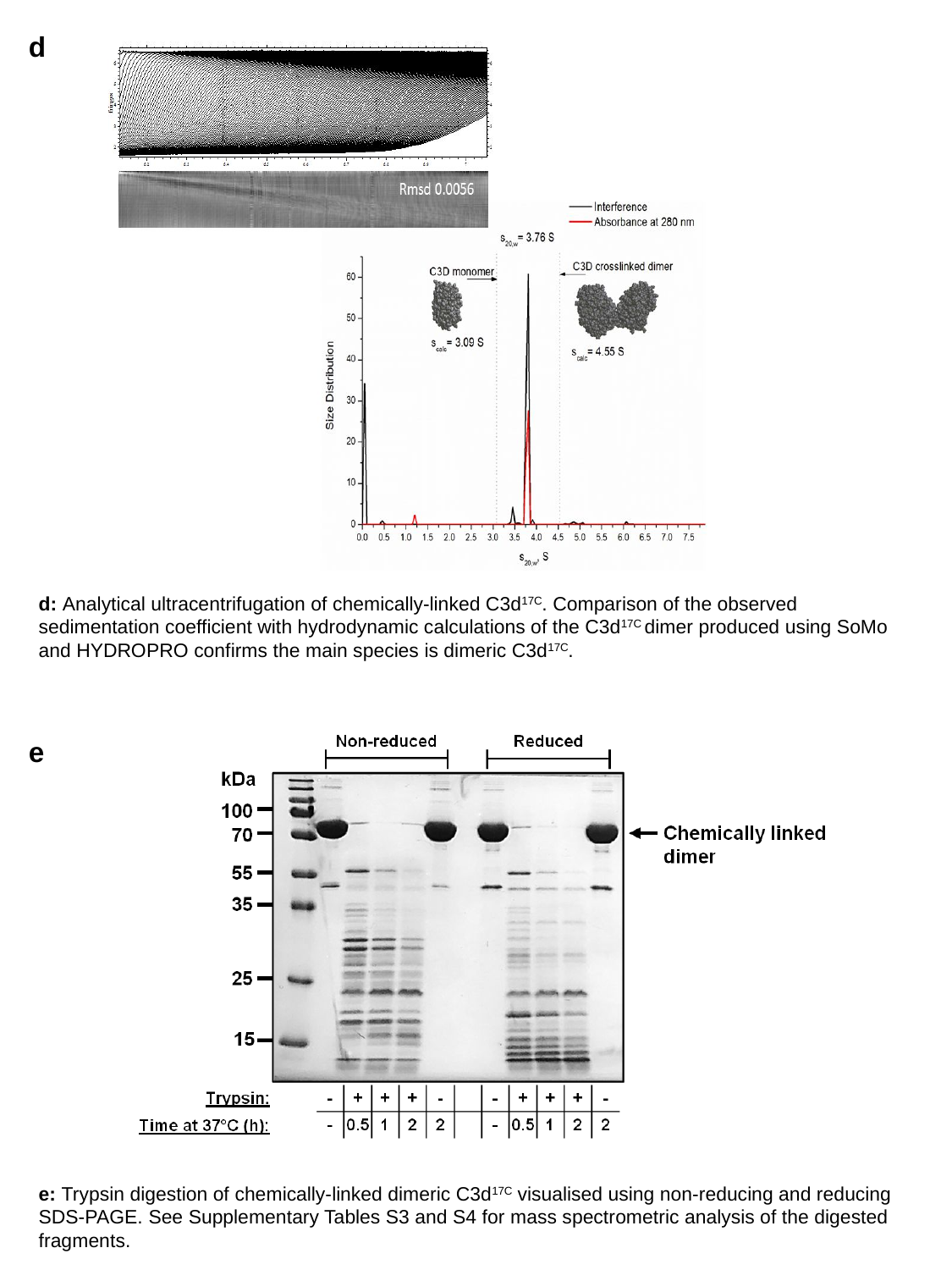

d
d: Analytical ultracentrifugation of chemically-linked C3d17C. Comparison of the observed sedimentation coefficient with hydrodynamic calculations of the C3d17C dimer produced using SoMo and HYDROPRO confirms the main species is dimeric C3d17C.
e
e: Trypsin digestion of chemically-linked dimeric C3d17C visualised using non-reducing and reducing SDS-PAGE. See Supplementary Tables S3 and S4 for mass spectrometric analysis of the digested fragments.

## Slide 15
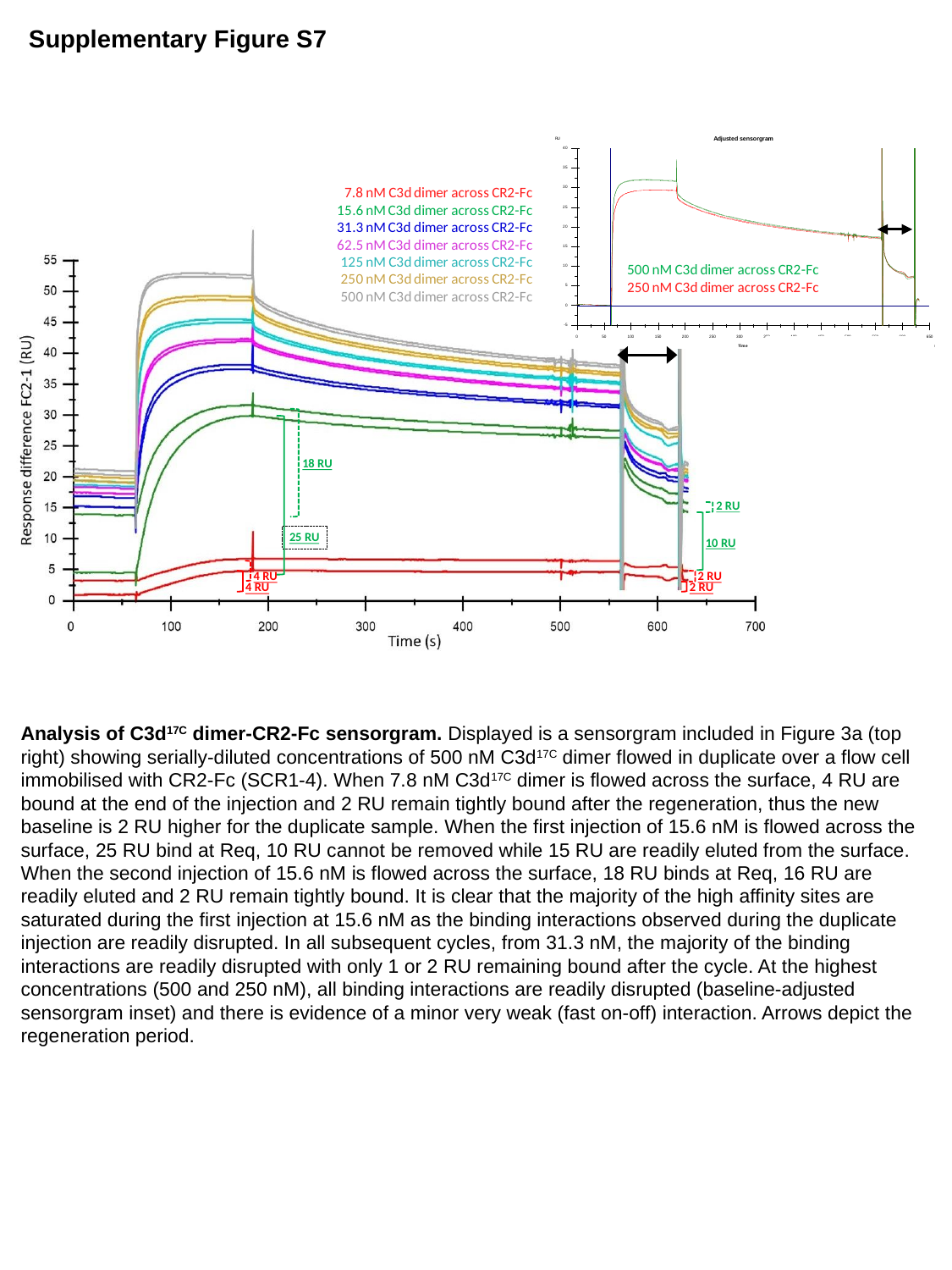

Supplementary Figure S7
Analysis of C3d17C dimer-CR2-Fc sensorgram. Displayed is a sensorgram included in Figure 3a (top right) showing serially-diluted concentrations of 500 nM C3d17C dimer flowed in duplicate over a flow cell immobilised with CR2-Fc (SCR1-4). When 7.8 nM C3d17C dimer is flowed across the surface, 4 RU are bound at the end of the injection and 2 RU remain tightly bound after the regeneration, thus the new baseline is 2 RU higher for the duplicate sample. When the first injection of 15.6 nM is flowed across the surface, 25 RU bind at Req, 10 RU cannot be removed while 15 RU are readily eluted from the surface. When the second injection of 15.6 nM is flowed across the surface, 18 RU binds at Req, 16 RU are readily eluted and 2 RU remain tightly bound. It is clear that the majority of the high affinity sites are saturated during the first injection at 15.6 nM as the binding interactions observed during the duplicate injection are readily disrupted. In all subsequent cycles, from 31.3 nM, the majority of the binding interactions are readily disrupted with only 1 or 2 RU remaining bound after the cycle. At the highest concentrations (500 and 250 nM), all binding interactions are readily disrupted (baseline-adjusted sensorgram inset) and there is evidence of a minor very weak (fast on-off) interaction. Arrows depict the regeneration period.

## Slide 16
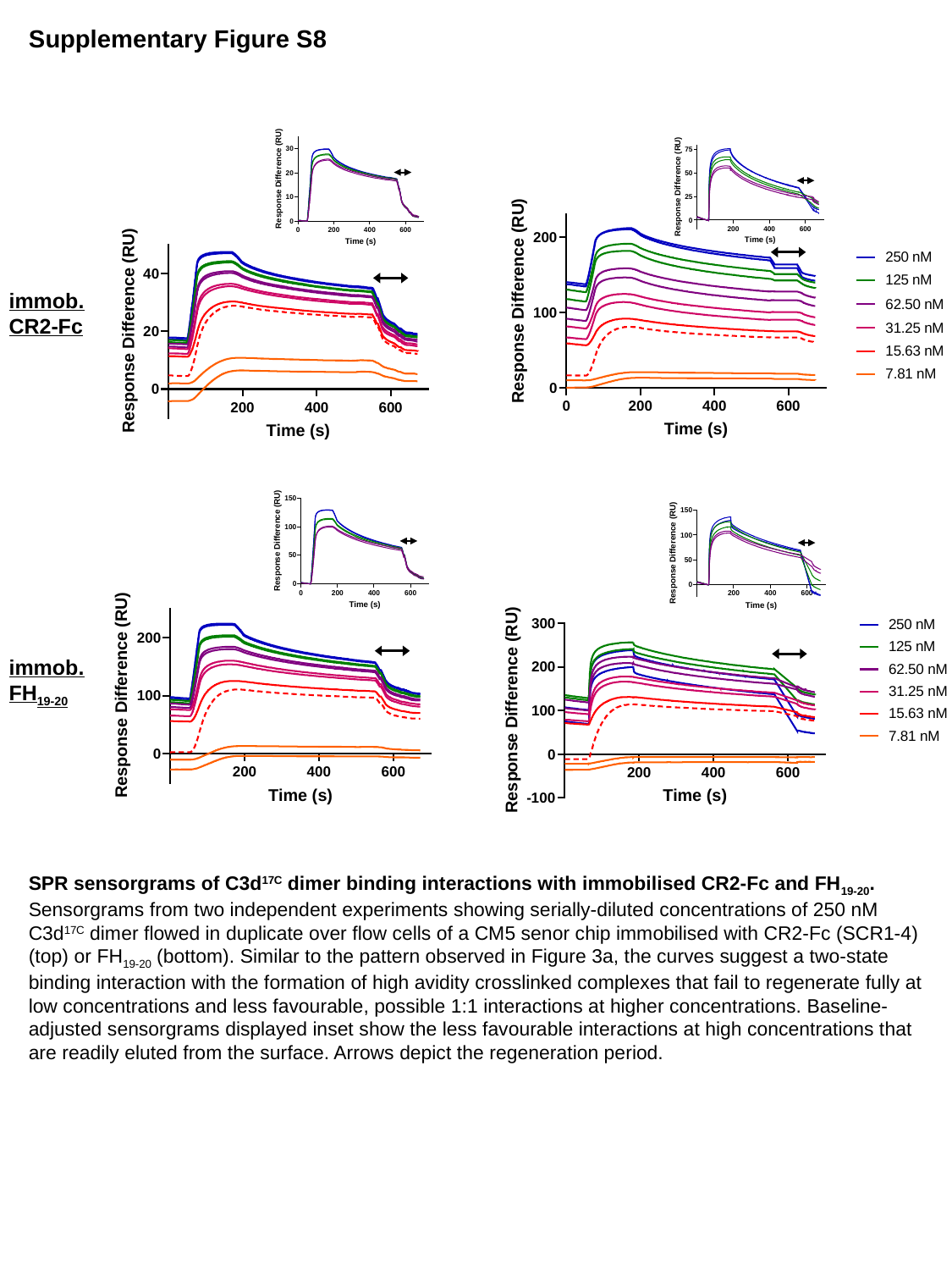

Supplementary Figure S8
immob.
CR2-Fc
immob.
FH19-20
SPR sensorgrams of C3d17C dimer binding interactions with immobilised CR2-Fc and FH19-20. Sensorgrams from two independent experiments showing serially-diluted concentrations of 250 nM C3d17C dimer flowed in duplicate over flow cells of a CM5 senor chip immobilised with CR2-Fc (SCR1-4) (top) or FH19-20 (bottom). Similar to the pattern observed in Figure 3a, the curves suggest a two-state binding interaction with the formation of high avidity crosslinked complexes that fail to regenerate fully at low concentrations and less favourable, possible 1:1 interactions at higher concentrations. Baseline-adjusted sensorgrams displayed inset show the less favourable interactions at high concentrations that are readily eluted from the surface. Arrows depict the regeneration period.

## Slide 17
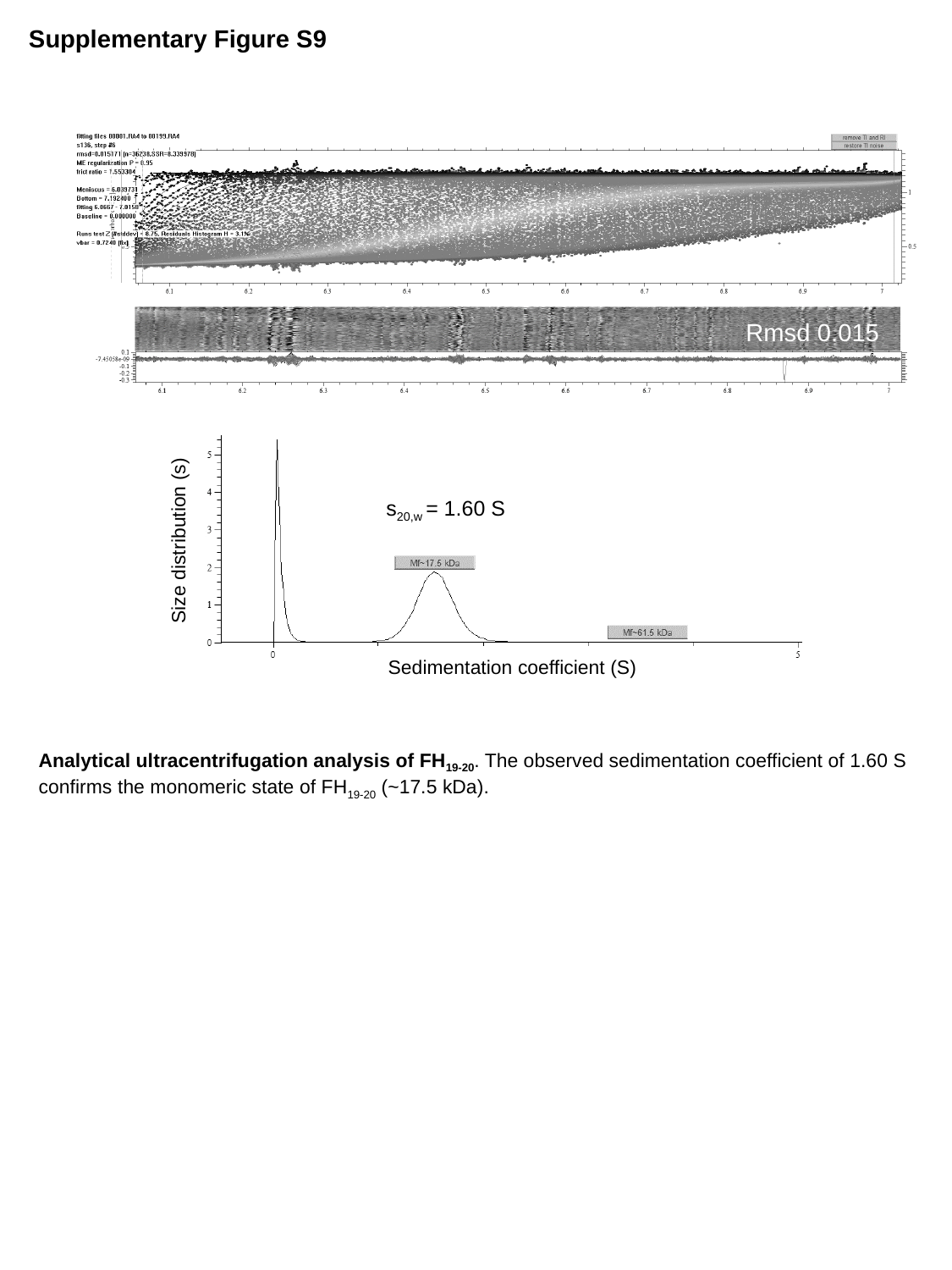

Supplementary Figure S9
Rmsd 0.015
s20,w = 1.60 S
Size distribution (s)
Sedimentation coefficient (S)
Analytical ultracentrifugation analysis of FH19-20. The observed sedimentation coefficient of 1.60 S confirms the monomeric state of FH19-20 (~17.5 kDa).

## Slide 18
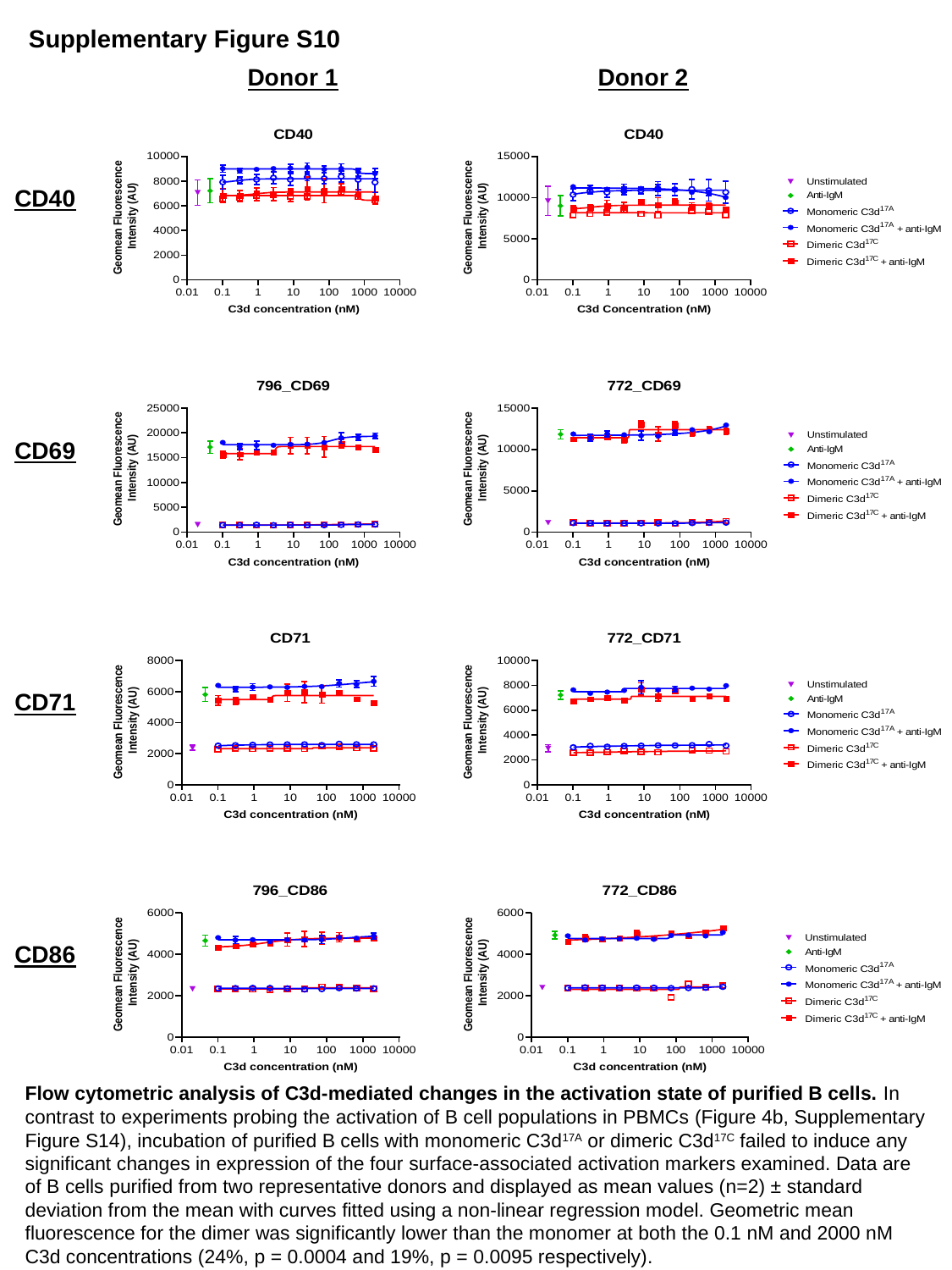

Supplementary Figure S10
Flow cytometric analysis of C3d-mediated changes in the activation state of purified B cells. In contrast to experiments probing the activation of B cell populations in PBMCs (Figure 4b, Supplementary Figure S14), incubation of purified B cells with monomeric C3d17A or dimeric C3d17C failed to induce any significant changes in expression of the four surface-associated activation markers examined. Data are of B cells purified from two representative donors and displayed as mean values (n=2) ± standard deviation from the mean with curves fitted using a non-linear regression model. Geometric mean fluorescence for the dimer was significantly lower than the monomer at both the 0.1 nM and 2000 nM C3d concentrations (24%, p = 0.0004 and 19%, p = 0.0095 respectively).

## Slide 19
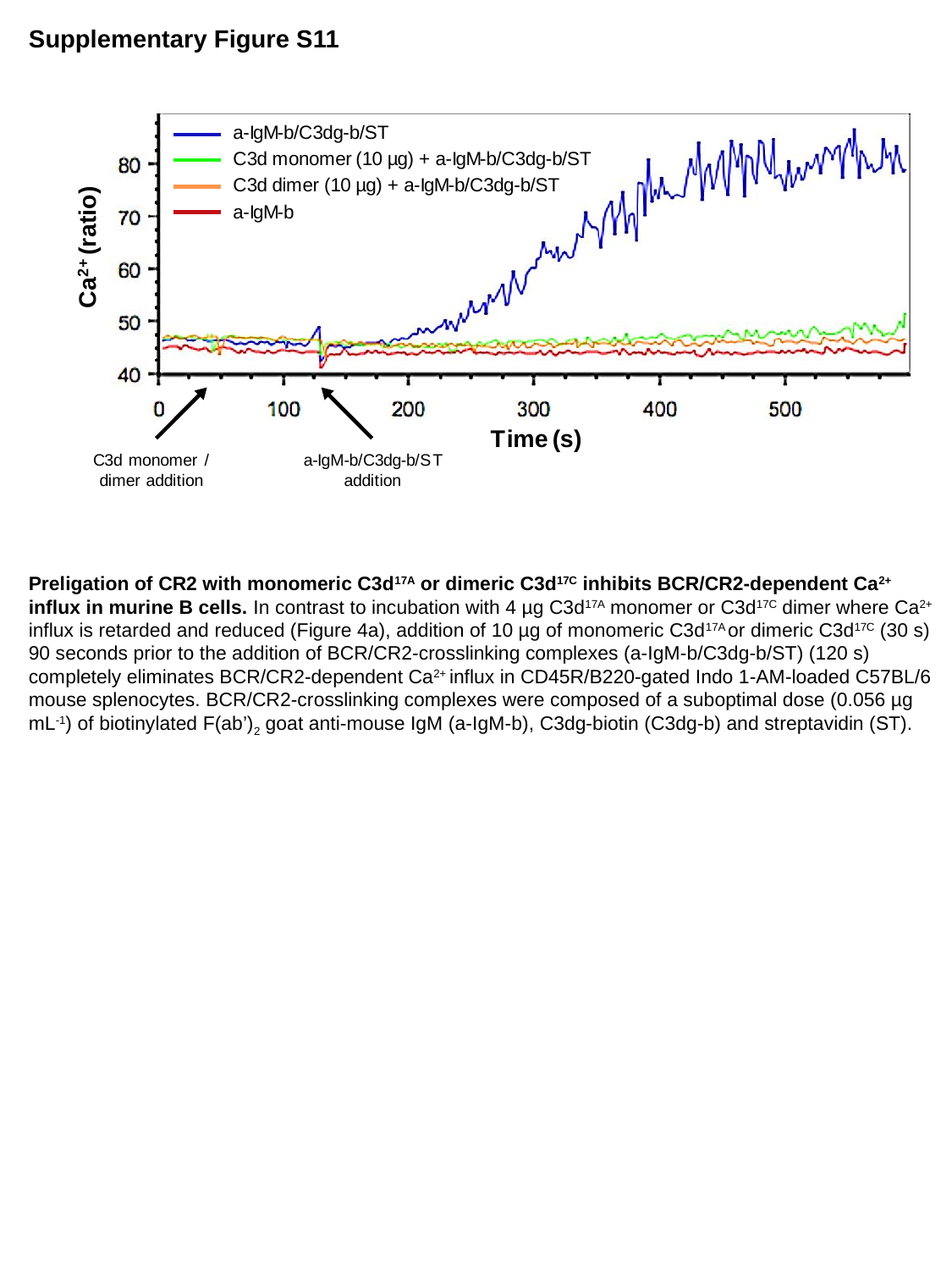

Supplementary Figure S11
Preligation of CR2 with monomeric C3d17A or dimeric C3d17C inhibits BCR/CR2-dependent Ca2+ influx in murine B cells. In contrast to incubation with 4 µg C3d17A monomer or C3d17C dimer where Ca2+ influx is retarded and reduced (Figure 4a), addition of 10 µg of monomeric C3d17A or dimeric C3d17C (30 s) 90 seconds prior to the addition of BCR/CR2-crosslinking complexes (a-IgM-b/C3dg-b/ST) (120 s) completely eliminates BCR/CR2-dependent Ca2+ influx in CD45R/B220-gated Indo 1-AM-loaded C57BL/6 mouse splenocytes. BCR/CR2-crosslinking complexes were composed of a suboptimal dose (0.056 µg mL-1) of biotinylated F(ab’)2 goat anti‐mouse IgM (a-IgM-b), C3dg-biotin (C3dg-b) and streptavidin (ST).

## Slide 20
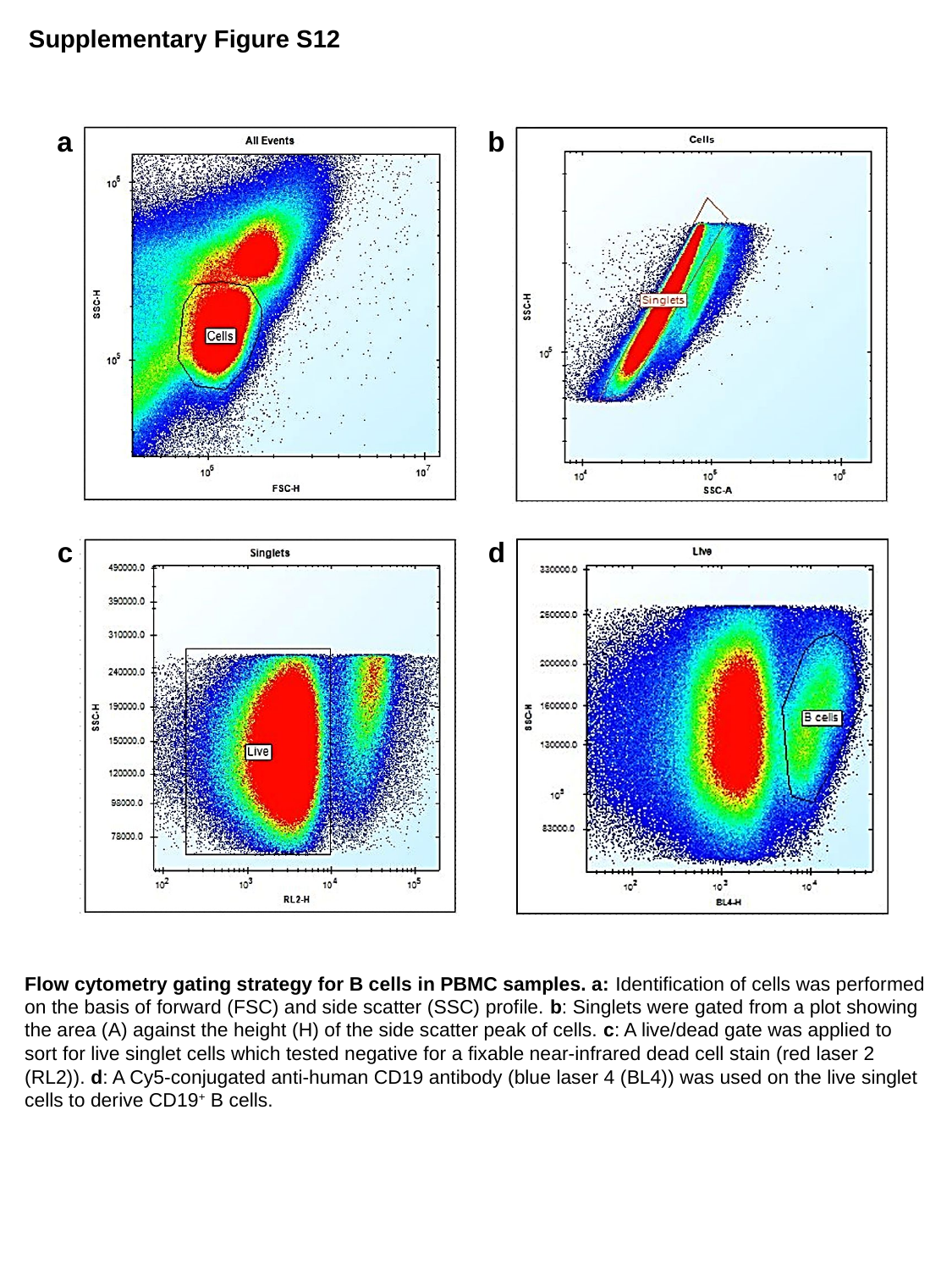

Supplementary Figure S12
a
b
c
d
Flow cytometry gating strategy for B cells in PBMC samples. a: Identification of cells was performed on the basis of forward (FSC) and side scatter (SSC) profile. b: Singlets were gated from a plot showing the area (A) against the height (H) of the side scatter peak of cells. c: A live/dead gate was applied to sort for live singlet cells which tested negative for a fixable near-infrared dead cell stain (red laser 2 (RL2)). d: A Cy5-conjugated anti-human CD19 antibody (blue laser 4 (BL4)) was used on the live singlet cells to derive CD19+ B cells.

## Slide 21
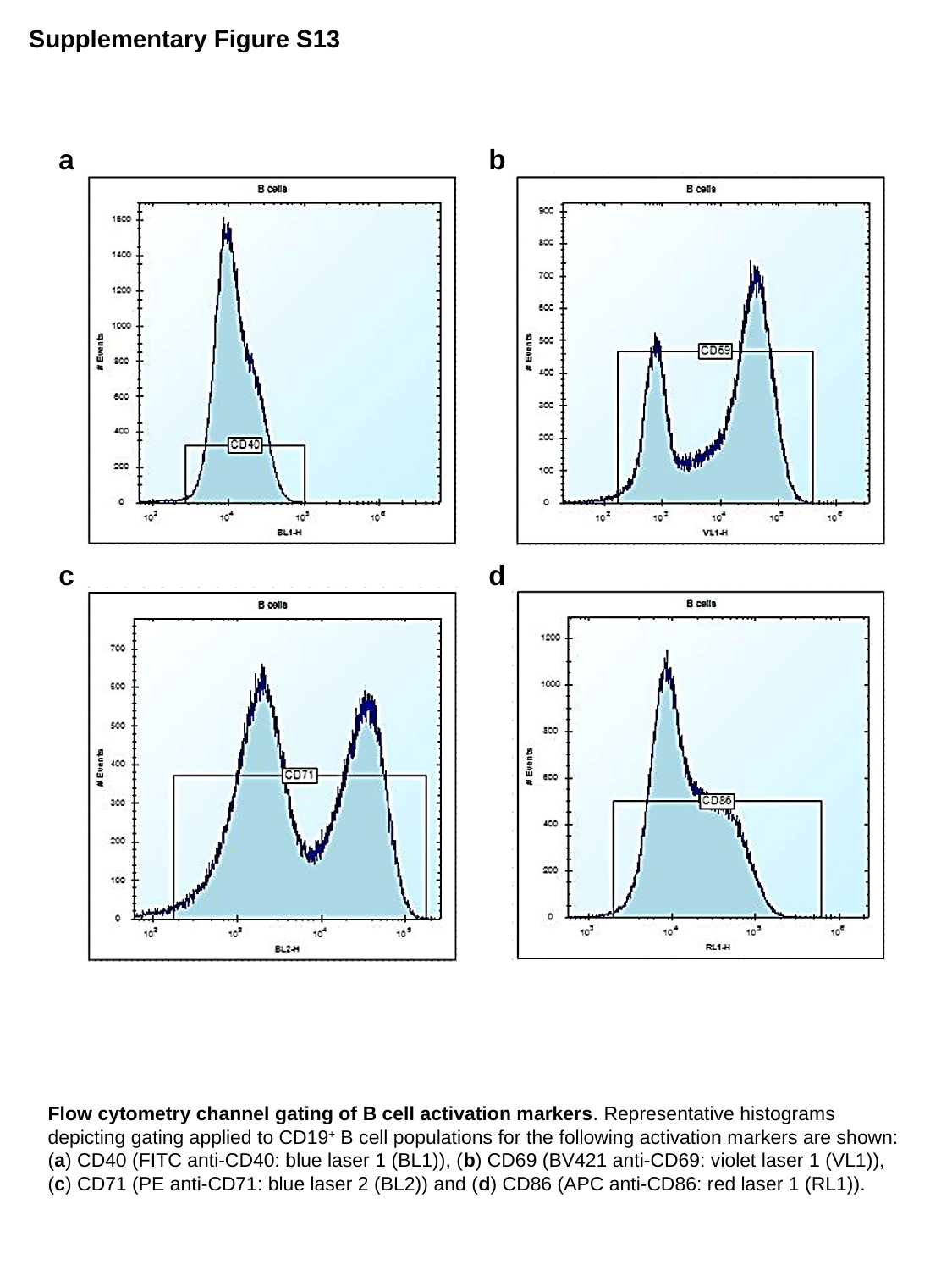

Supplementary Figure S13
a
b
c
d
Flow cytometry channel gating of B cell activation markers. Representative histograms depicting gating applied to CD19+ B cell populations for the following activation markers are shown: (a) CD40 (FITC anti-CD40: blue laser 1 (BL1)), (b) CD69 (BV421 anti-CD69: violet laser 1 (VL1)), (c) CD71 (PE anti-CD71: blue laser 2 (BL2)) and (d) CD86 (APC anti-CD86: red laser 1 (RL1)).

## Slide 22
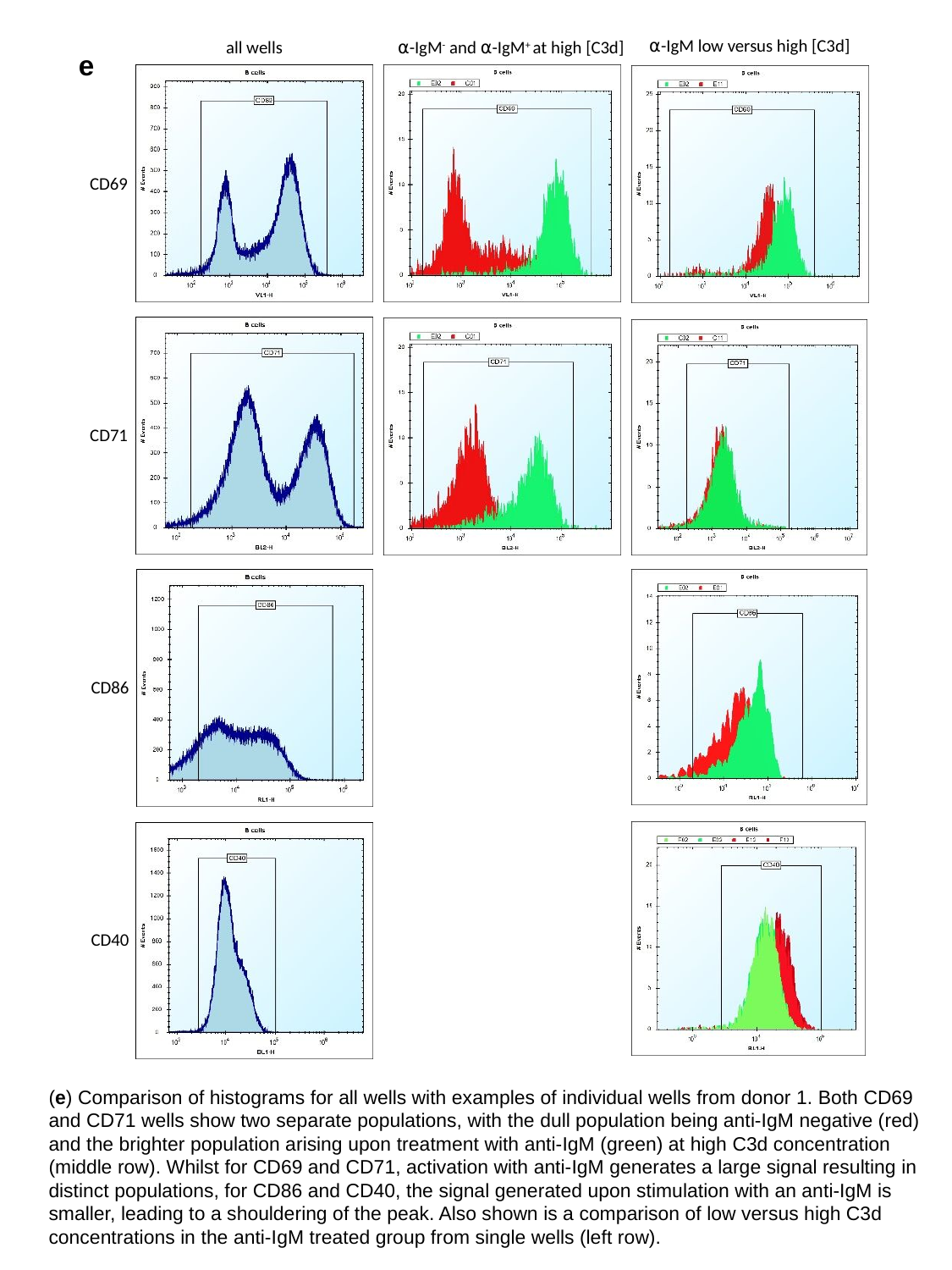

⍺-IgM low versus high [C3d]
all wells
⍺-IgM- and ⍺-IgM+ at high [C3d]
e
CD69
CD71
CD86
CD40
(e) Comparison of histograms for all wells with examples of individual wells from donor 1. Both CD69 and CD71 wells show two separate populations, with the dull population being anti-IgM negative (red) and the brighter population arising upon treatment with anti-IgM (green) at high C3d concentration (middle row). Whilst for CD69 and CD71, activation with anti-IgM generates a large signal resulting in distinct populations, for CD86 and CD40, the signal generated upon stimulation with an anti-IgM is smaller, leading to a shouldering of the peak. Also shown is a comparison of low versus high C3d concentrations in the anti-IgM treated group from single wells (left row).

## Slide 23
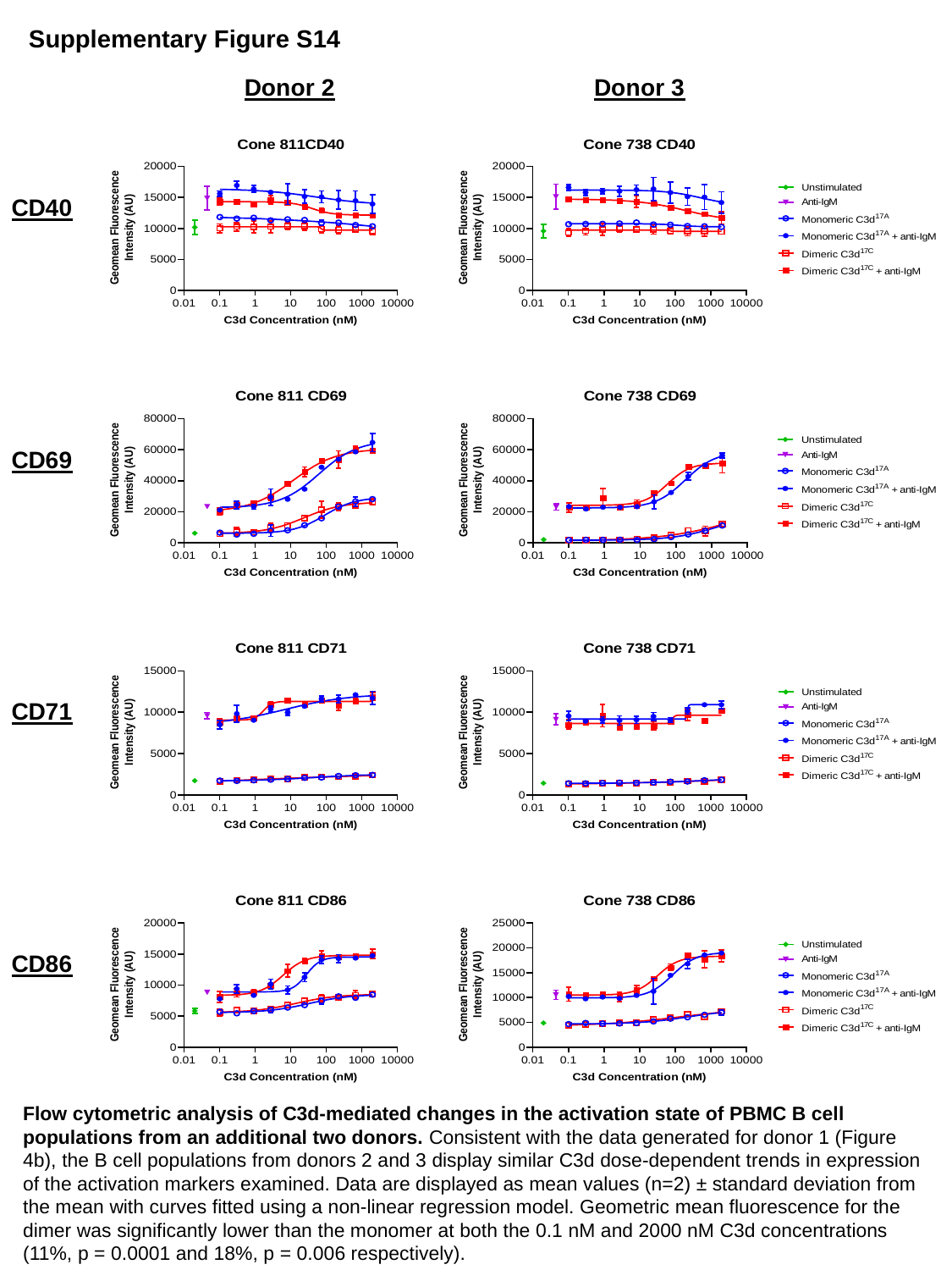

Supplementary Figure S14
Flow cytometric analysis of C3d-mediated changes in the activation state of PBMC B cell populations from an additional two donors. Consistent with the data generated for donor 1 (Figure 4b), the B cell populations from donors 2 and 3 display similar C3d dose-dependent trends in expression of the activation markers examined. Data are displayed as mean values (n=2) ± standard deviation from the mean with curves fitted using a non-linear regression model. Geometric mean fluorescence for the dimer was significantly lower than the monomer at both the 0.1 nM and 2000 nM C3d concentrations (11%, p = 0.0001 and 18%, p = 0.006 respectively).

## Slide 24
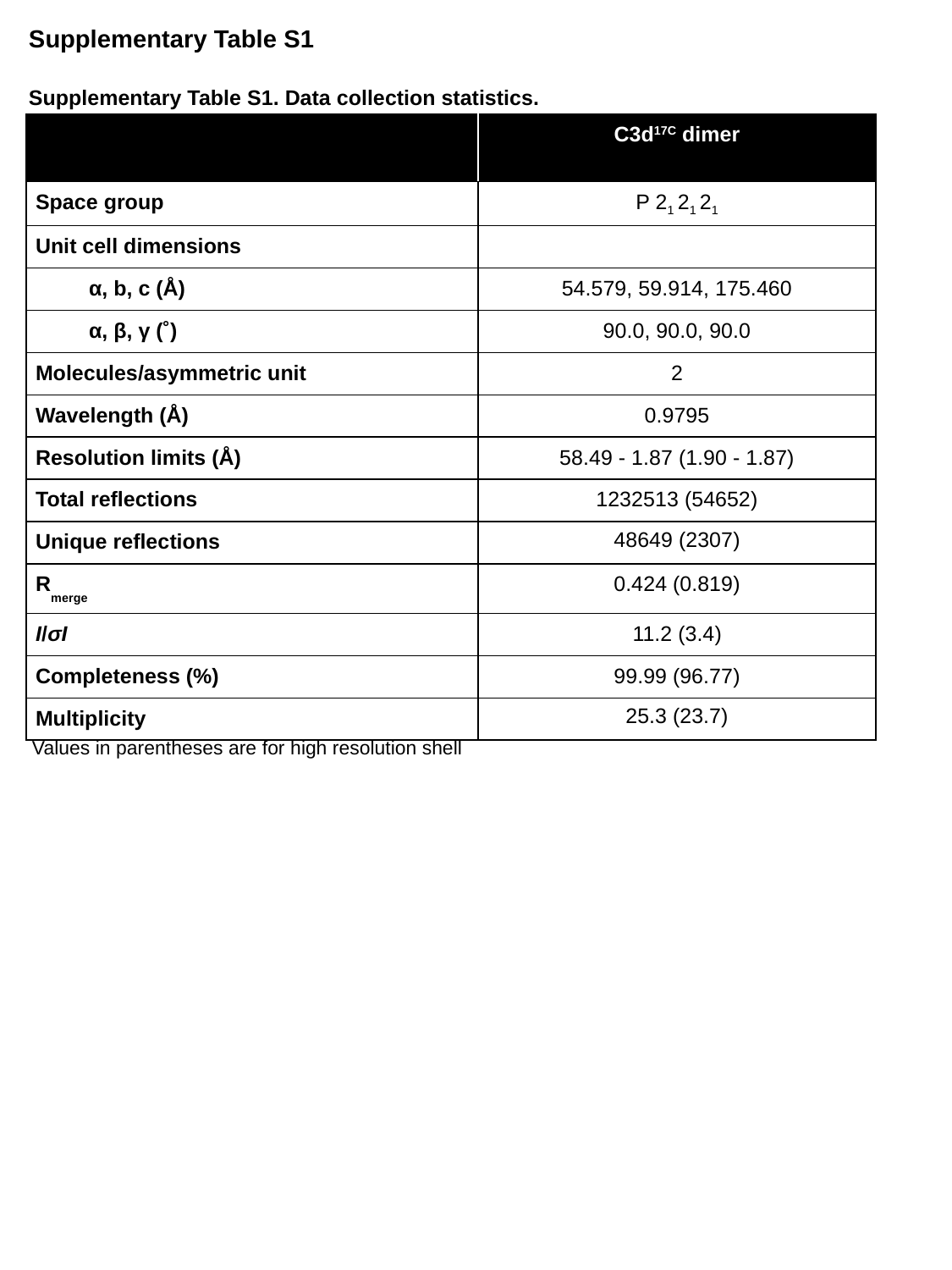

Supplementary Table S1
Supplementary Table S1. Data collection statistics.
| | C3d17C dimer |
| --- | --- |
| Space group | P 21 21 21 |
| Unit cell dimensions | |
| α, b, c (Å) | 54.579, 59.914, 175.460 |
| α, β, γ (˚) | 90.0, 90.0, 90.0 |
| Molecules/asymmetric unit | 2 |
| Wavelength (Å) | 0.9795 |
| Resolution limits (Å) | 58.49 - 1.87 (1.90 - 1.87) |
| Total reflections | 1232513 (54652) |
| Unique reflections | 48649 (2307) |
| Rmerge | 0.424 (0.819) |
| I/σI | 11.2 (3.4) |
| Completeness (%) | 99.99 (96.77) |
| Multiplicity | 25.3 (23.7) |
Values in parentheses are for high resolution shell

## Slide 25
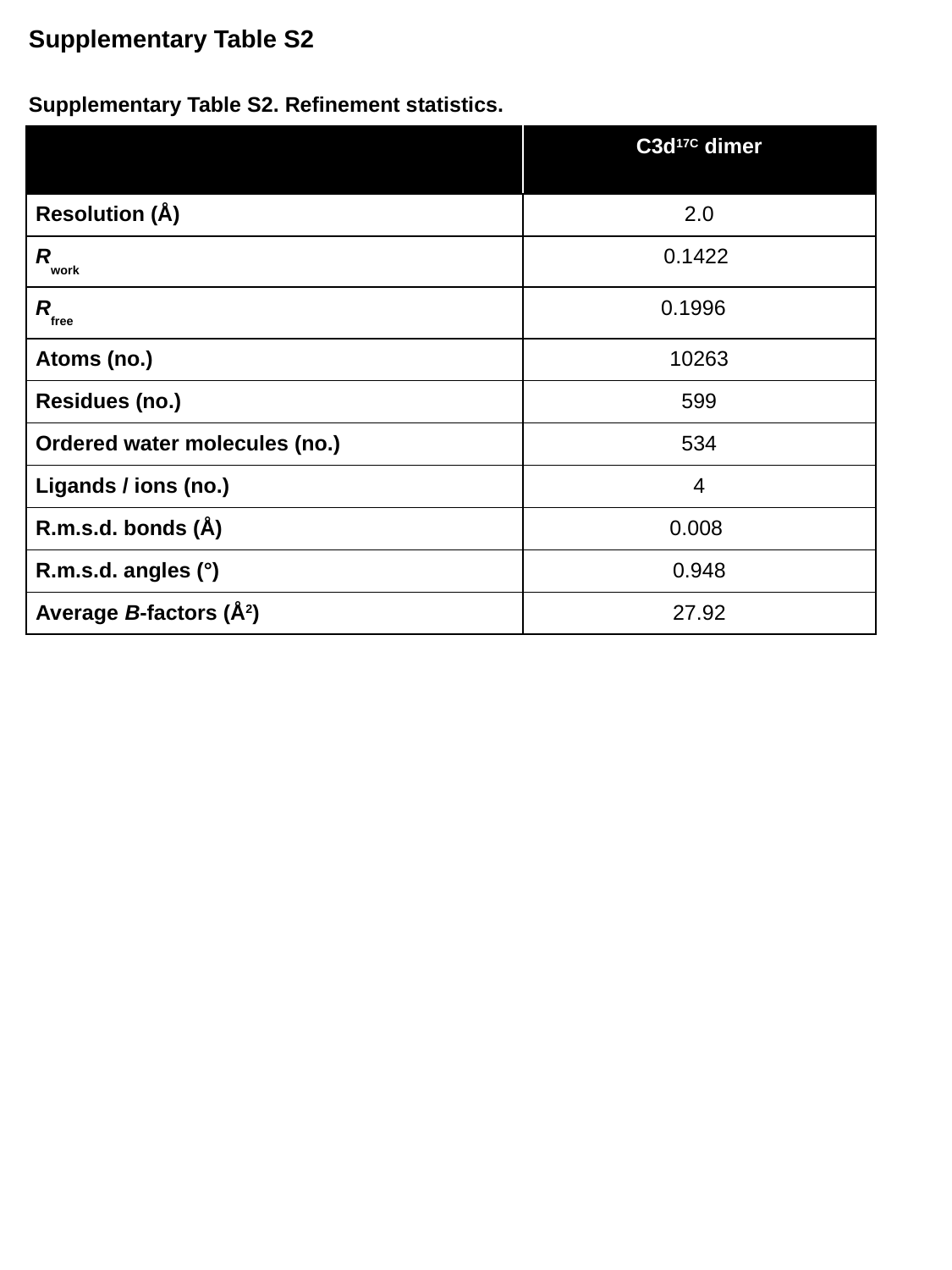

Supplementary Table S2
Supplementary Table S2. Refinement statistics.
| | C3d17C dimer |
| --- | --- |
| Resolution (Å) | 2.0 |
| Rwork | 0.1422 |
| Rfree | 0.1996 |
| Atoms (no.) | 10263 |
| Residues (no.) | 599 |
| Ordered water molecules (no.) | 534 |
| Ligands / ions (no.) | 4 |
| R.m.s.d. bonds (Å) | 0.008 |
| R.m.s.d. angles (°) | 0.948 |
| Average B-factors (Å2) | 27.92 |

## Slide 26
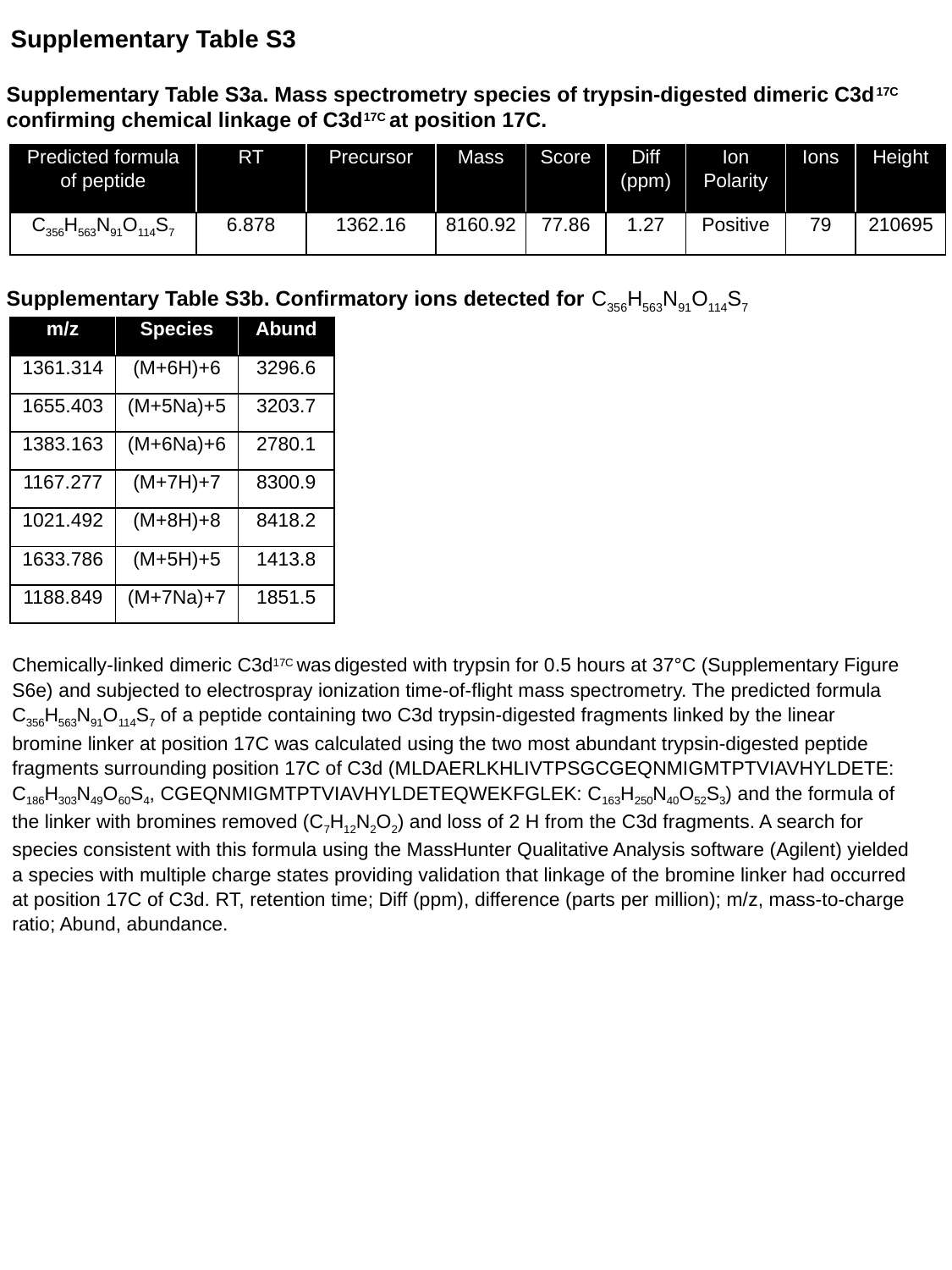

Supplementary Table S3
Supplementary Table S3a. Mass spectrometry species of trypsin-digested dimeric C3d17C confirming chemical linkage of C3d17C at position 17C.
| Predicted formula of peptide | RT | Precursor | Mass | Score | Diff (ppm) | Ion Polarity | Ions | Height |
| --- | --- | --- | --- | --- | --- | --- | --- | --- |
| C356H563N91O114S7 | 6.878 | 1362.16 | 8160.92 | 77.86 | 1.27 | Positive | 79 | 210695 |
Supplementary Table S3b. Confirmatory ions detected for C356H563N91O114S7
| m/z | Species | Abund |
| --- | --- | --- |
| 1361.314 | (M+6H)+6 | 3296.6 |
| 1655.403 | (M+5Na)+5 | 3203.7 |
| 1383.163 | (M+6Na)+6 | 2780.1 |
| 1167.277 | (M+7H)+7 | 8300.9 |
| 1021.492 | (M+8H)+8 | 8418.2 |
| 1633.786 | (M+5H)+5 | 1413.8 |
| 1188.849 | (M+7Na)+7 | 1851.5 |
Chemically-linked dimeric C3d17C was digested with trypsin for 0.5 hours at 37°C (Supplementary Figure S6e) and subjected to electrospray ionization time-of-flight mass spectrometry. The predicted formula C356H563N91O114S7 of a peptide containing two C3d trypsin-digested fragments linked by the linear bromine linker at position 17C was calculated using the two most abundant trypsin-digested peptide fragments surrounding position 17C of C3d (MLDAERLKHLIVTPSGCGEQNMIGMTPTVIAVHYLDETE: C186H303N49O60S4, CGEQNMIGMTPTVIAVHYLDETEQWEKFGLEK: C163H250N40O52S3) and the formula of the linker with bromines removed (C7H12N2O2) and loss of 2 H from the C3d fragments. A search for species consistent with this formula using the MassHunter Qualitative Analysis software (Agilent) yielded a species with multiple charge states providing validation that linkage of the bromine linker had occurred at position 17C of C3d. RT, retention time; Diff (ppm), difference (parts per million); m/z, mass-to-charge ratio; Abund, abundance.

## Slide 27
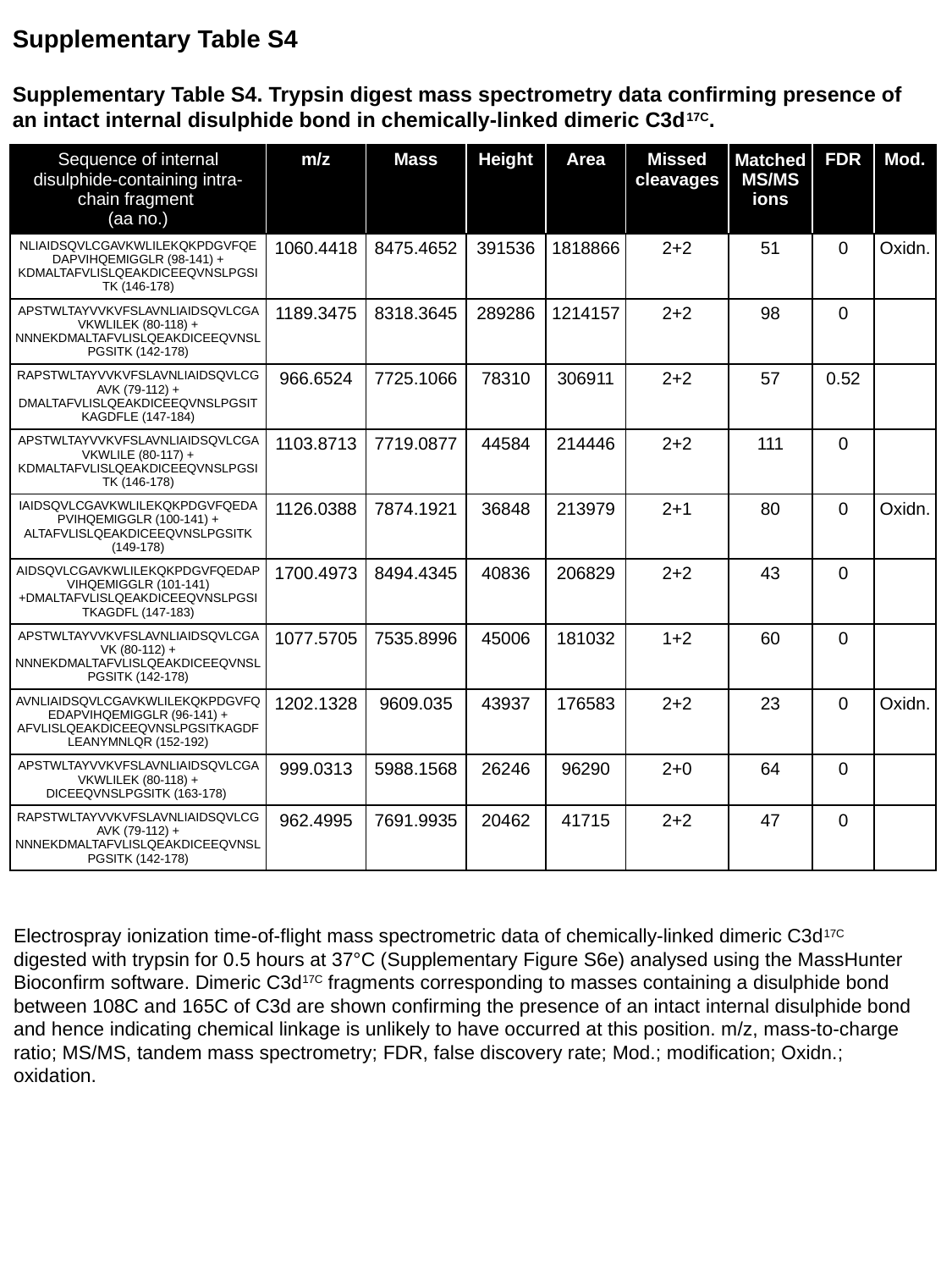

Supplementary Table S4
Supplementary Table S4. Trypsin digest mass spectrometry data confirming presence of an intact internal disulphide bond in chemically-linked dimeric C3d17C.
| Sequence of internal disulphide-containing intra-chain fragment (aa no.) | m/z | Mass | Height | Area | Missed cleavages | Matched MS/MS ions | FDR | Mod. |
| --- | --- | --- | --- | --- | --- | --- | --- | --- |
| NLIAIDSQVLCGAVKWLILEKQKPDGVFQEDAPVIHQEMIGGLR (98-141) + KDMALTAFVLISLQEAKDICEEQVNSLPGSITK (146-178) | 1060.4418 | 8475.4652 | 391536 | 1818866 | 2+2 | 51 | 0 | Oxidn. |
| APSTWLTAYVVKVFSLAVNLIAIDSQVLCGAVKWLILEK (80-118) + NNNEKDMALTAFVLISLQEAKDICEEQVNSLPGSITK (142-178) | 1189.3475 | 8318.3645 | 289286 | 1214157 | 2+2 | 98 | 0 | |
| RAPSTWLTAYVVKVFSLAVNLIAIDSQVLCGAVK (79-112) + DMALTAFVLISLQEAKDICEEQVNSLPGSITKAGDFLE (147-184) | 966.6524 | 7725.1066 | 78310 | 306911 | 2+2 | 57 | 0.52 | |
| APSTWLTAYVVKVFSLAVNLIAIDSQVLCGAVKWLILE (80-117) + KDMALTAFVLISLQEAKDICEEQVNSLPGSITK (146-178) | 1103.8713 | 7719.0877 | 44584 | 214446 | 2+2 | 111 | 0 | |
| IAIDSQVLCGAVKWLILEKQKPDGVFQEDAPVIHQEMIGGLR (100-141) + ALTAFVLISLQEAKDICEEQVNSLPGSITK (149-178) | 1126.0388 | 7874.1921 | 36848 | 213979 | 2+1 | 80 | 0 | Oxidn. |
| AIDSQVLCGAVKWLILEKQKPDGVFQEDAPVIHQEMIGGLR (101-141) +DMALTAFVLISLQEAKDICEEQVNSLPGSITKAGDFL (147-183) | 1700.4973 | 8494.4345 | 40836 | 206829 | 2+2 | 43 | 0 | |
| APSTWLTAYVVKVFSLAVNLIAIDSQVLCGAVK (80-112) + NNNEKDMALTAFVLISLQEAKDICEEQVNSLPGSITK (142-178) | 1077.5705 | 7535.8996 | 45006 | 181032 | 1+2 | 60 | 0 | |
| AVNLIAIDSQVLCGAVKWLILEKQKPDGVFQEDAPVIHQEMIGGLR (96-141) + AFVLISLQEAKDICEEQVNSLPGSITKAGDFLEANYMNLQR (152-192) | 1202.1328 | 9609.035 | 43937 | 176583 | 2+2 | 23 | 0 | Oxidn. |
| APSTWLTAYVVKVFSLAVNLIAIDSQVLCGAVKWLILEK (80-118) + DICEEQVNSLPGSITK (163-178) | 999.0313 | 5988.1568 | 26246 | 96290 | 2+0 | 64 | 0 | |
| RAPSTWLTAYVVKVFSLAVNLIAIDSQVLCGAVK (79-112) + NNNEKDMALTAFVLISLQEAKDICEEQVNSLPGSITK (142-178) | 962.4995 | 7691.9935 | 20462 | 41715 | 2+2 | 47 | 0 | |
Electrospray ionization time-of-flight mass spectrometric data of chemically-linked dimeric C3d17C digested with trypsin for 0.5 hours at 37°C (Supplementary Figure S6e) analysed using the MassHunter Bioconfirm software. Dimeric C3d17C fragments corresponding to masses containing a disulphide bond between 108C and 165C of C3d are shown confirming the presence of an intact internal disulphide bond and hence indicating chemical linkage is unlikely to have occurred at this position. m/z, mass-to-charge ratio; MS/MS, tandem mass spectrometry; FDR, false discovery rate; Mod.; modification; Oxidn.; oxidation.
